# Clinically conserved genomic subtypes of gastric adenocarcinoma

**DOI:** 10.1186/s12943-023-01796-w

**Published:** 2023-09-06

**Authors:** Yun Seong Jeong, Young-Gyu Eun, Sung Hwan Lee, Sang-Hee Kang, Sun Young Yim, Eui Hyun Kim, Joo Kyung Noh, Bo Hwa Sohn, Seon Rang Woo, Moonkyoo Kong, Deok Hwa Nam, Hee-Jin Jang, Hyun-Sung Lee, Shumei Song, Sang Cheul Oh, Jeeyun Lee, Jaffer A. Ajani, Ju-Seog Lee

**Affiliations:** 1 Department of Systems Biology, The University of Texas MD Anderson Cancer Center, 1515 Holcombe Blvd., Unit 1058, Houston, TX 77030 USA; 2 Department of Biomedical Science and Technology, Graduate School, Kyung Hee University, Seoul, Korea; 3 Department of Otolaryngology - Head and Neck Surgery, Kyung Hee University Medical Center, Kyung Hee University School of Medicine, Seoul, Korea; 4 Division of Hepatobiliary and Pancreatic Surgery, Department of Surgery, Yonsei University College of Medicine, Seoul, Korea; 5 Division of Hepatobiliary and Pancreas, Department of Surgery, CHA Bundang Medical Center, CHA University, Pocheon, Korea; 6 Department of Surgery, Korea University Guro Hospital, Seoul, Korea; 7 Division of Gastroenterology and Hepatology, Department of Internal Medicine, Korea University College of Medicine, Seoul, Korea; 8 Department of Neurosurgery, Yonsei University College of Medicine, Seoul, Korea; 9 Department of Radiation Oncology, Kyung Hee University Medical Center, Kyung Hee University School of Medicine, Seoul, Korea; 10 Systems Onco-Immunology Laboratory, David J. Sugarbaker Division of Thoracic Surgery, Michael E. DeBakey Department of Surgery, Baylor College of Medicine, Houston, TX USA; 11 Department of Gastrointestinal Medical Oncology, The University of Texas MD Anderson Cancer Center, Houston, TX USA; 12 Division of Oncology/Hematology, Department of Internal Medicine, Korea University College of Medicine, Seoul, Korea; 13 Division of Hematology-Oncology, Department of Medicine, Samsung Medical Center, Sungkyunkwan University School of Medicine, Seoul, Korea

**Keywords:** Gastric cancer, Consensus subtype, Clinical subtypes, Stem cells, Cancer immune activity, Radiation therapy

## Abstract

Gastric adenocarcinoma (GAC) is a lethal disease characterized by genomic and clinical heterogeneity. By integrating 8 previously established genomic signatures for GAC subtypes, we identified 6 clinically and molecularly distinct genomic consensus subtypes (CGSs). CGS1 have the poorest prognosis, very high stem cell characteristics, and high IGF1 expression, but low genomic alterations. CGS2 is enriched with canonical epithelial gene expression. CGS3 and CGS4 have high copy number alterations and low immune reactivity. However, CGS3 and CGS4 differ in that CGS3 has high HER2 activation, while CGS4 has high SALL4 and KRAS activation. CGS5 has the high mutation burden and moderately high immune reactivity that are characteristic of microsatellite instable tumors. Most CGS6 tumors are positive for Epstein Barr virus and show extremely high levels of methylation and high immune reactivity. In a systematic analysis of genomic and proteomic data, we estimated the potential response rate of each consensus subtype to standard and experimental treatments such as radiation therapy, targeted therapy, and immunotherapy. Interestingly, CGS3 was significantly associated with a benefit from chemoradiation therapy owing to its high basal level of ferroptosis. In addition, we also identified potential therapeutic targets for each consensus subtype. Thus, the consensus subtypes produced a robust classification and provide for additional characterizations for subtype-based customized interventions.

**Supplementary Information:**

The online version contains supplementary material available at 10.1186/s12943-023-01796-w.

## Background

Gastric adenocarcinoma (GAC) is the fifth most commonly diagnosed cancer and the third leading cause of cancer death in the world, following only lung and colorectal cancers in overall mortality [1]. Despite recent advances in treatment including immunotherapy, the prognosis for patients with advanced GAC remains poor [2]. The development of GAC is a multistep process driven by genomic and epigenomic alterations that lead to the transformation of epithelial cells in the stomach and then the growth and invasion of cancer cells [3]. GAC is a highly heterogeneous disease, as reflected by its wide range of clinical outcomes in terms of overall survival, early recurrence, and resistance to treatment [4]. Therefore, a robust classification of GACs into clinically and molecularly homogeneous subgroups could significantly improve treatment selection. Many investigators have devoted considerable effort in establishing such classifications by various approaches. However, there are still considerable gaps in translating molecular subtypes into clinical practice.

The clinical relevance of genomic and molecular subtypes of GAC has been demonstrated by several studies showing their significant association with overall survival, early recurrence, and treatment response [5]. However, their translation to the clinic has been hindered by discrepancies in classification methods that might be attributable to ethnic differences of patients, platforms used for collecting genomic data, sample collection and processing methods, and the prediction algorithms used. Despite these discrepancies, there is substantial overlap among the subtypes identified previously. For example, the mesenchymal phenotype (MP) identified in the MD Anderson Cancer Center study is highly similar to the genome stable (GS) subtype of The Cancer Genome Atlas (TCGA) study, the mesenchymal subtype of the Yonsei Cancer Center study, and the microsatellite stable/epithelial-to-mesenchymal transition (MSS/EMT) subtype of the Asian Cancer Research Group (ACRG) study [6–9]. Similarly, the Epstein Barr virus (EBV) subtype in the TCGA study is similar to the microsatellite instable/p53 + (MSI/p53 +) subtype of the ACRG study. Therefore, finding the consensus GAC subtypes would be highly useful for uncovering shared oncogenic/immunogenic molecular alterations that would lead to better selection of therapeutic targets and identification of robust biomarkers for predicting prognosis or treatment response. To address this need, we performed a meta-analysis of previously identified GAC subtypes and uncovered clinically distinct genomic subtypes.

## Methods

### Gene expression data from gastric cancers

TCGA RNA sequencing data for GAC was downloaded from the University of California, Santa Cruz, Genomics Institute on July 11, 2017 (https://xenabrowser.net/) [6]. RSEM-normalized data were log-transformed. Gene expression data from the Korea cohort were generated in earlier studies [7, 10, 11]; the data are publicly available from the NCBI’s GEO database (GSE26899, GSE2690, and GSE13861). Gene expression data from the Samsung Medical Center (GSE66229) [12], ACRG (GSE66229) [9], Shanxi Hospital (GSE29272) [13], Ruijin Hospital (GSE54129), Singapore1 (GSE29998) [14], Singapore2 (GSE15459) [15], KNCC (GSE14208, GSE14209) [16], and Yonsei Hospital (GSE84437) [8] cohorts have been described previously and are available from the NCBI’s GEO database.

### Clinically defined gastric cancer subtypes and associated gene expression signatures

The intrinsic subtype of gastric cancer [17], Hippo pathway subtypes [18], TCGA4 subtype [6, 11], TCGA risk score [11], ACRG subtype [9], Yonsei subtype [8], mesenchymal subtype [7], and lncRNA subtypes [19] and their associated gene signatures were described in earlier studies as listed in Supplementary Table S2.

### Statistical analysis of genomic and clinical data

The BRB Array Tools software program (http://linus.nci.nih.gov/BRB-ArrayTools.html) was used for analysis of the gene expression data and construction of a prediction model [20]. A heat map was generated using the Cluster and TreeView software programs [21], and further statistical analysis was performed using the R language (http://www.r-project.org).

### Classification of tumors and cluster of clusters approach (COCA)

Gastric tumors in 6 discovery cohorts were stratified into subtypes according to the prediction models and algorithms used in the original studies by using the BRB Array Tools software program. Before gene expression data in the discovery sets were collated for the construction of the prediction models, the expression levels of genes in each data set were independently standardized by transforming each gene’s expression level to a mean of 0 and a standard deviation of 1, as described previously [22, 23].

**Fig. 1:**
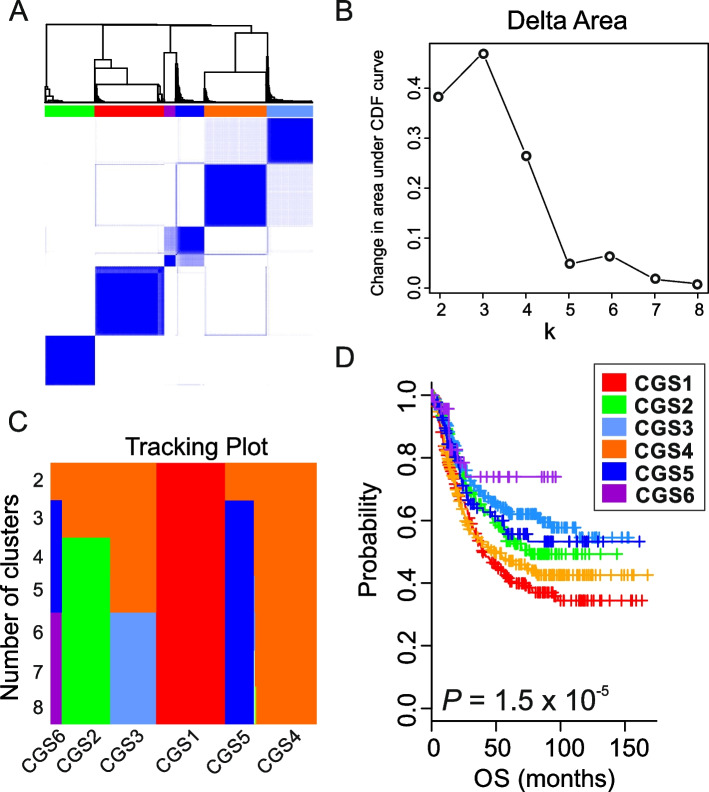
Consensus subtypes of gastric adenocarcinoma discovered by cluster-of-cluster assignment. **A** Hierarchical dendrogram of supercluster subtypes identified by cluster-of-cluster assignment (COCA). GAC tumors were grouped according to shared features of independently discovered molecular subtypes. Tumors from 6 GAC cohorts (Korea, Samsung, TCGA, Shanghai, Shanxi, and Singapore1, *n* = 1427) were stratified by 8 genomic classification methods, and subtype information was used for clustering with ConsensusClusterPlus (v.3.12). Columns and rows represent GAC tumors in the discovery/training set. **B** Delta area plot. The plot shows the relative change in area under the cumulative distribution function (CDF) curve comparing k and k − 1. **C** Tracking plot. The plot shows the cluster assignment of tumors (columns) for each k (rows) by color. This plot provides a view of item cluster membership across different values of k. **D** Clinical significance of 6 consensus subtypes of GAC in discovery/training cohorts (*n* = 1185). Patients in the Korea, TCGA, Shanxi, and Samsung cohorts were included in the analysis; clinical data from the Shanghai and Singapore1 cohorts were not available. OS, overall survival. *P*-value indicates the significance from log-rank test

A COCA was used to find consensus subtypes of gastric tumors as described previously [24–27]. The advantage of this approach is that it could combine data from across the 8 classification methods without the need for normalization steps prior to clustering, and each classification method influenced the final integrated result with a weight proportional to the number of subtypes. Briefly, the algorithm takes as input the binary vectors (1 or 0) that represent each of the genomic subtypes and re-clusters the samples according to those vectors by using ConsensusClusterPlus algorithm (v.3.12) [28]. On the basis of delta are plot and tracking plot of consensus clustering, k = 6 is selected for optimal number of subgroups (Fig. 1). Relationships between consensus subtypes and previous classifications were visualized using Circos [29].

### Analysis of genomic and proteomic data

Somatic mutations and indel (insertion and deletion) calls from exome sequencing of GAC tumors and copy number alteration data were obtained from cbioportal (http://www.cbioportal.org/). Silent mutations were not included. Gene expression data and copy number data were available for 384 tumors.

For characterization of the 6 consensus subtypes by DNA methylation status, we used level 3 β-values from the Illumina Infinium HumanMethylation450 Array platform. The data set consisted of 377 tumors. To identify subtype-specific methylation patterns, we performed differential methylation analyses on the basis of 2-sample *t*-tests, comparing each consensus subtype with the other subtypes.

For miRNA characterization of the 6 consensus subtypes, we obtained level 3 reads per million miRNAs mapped (RPM) data from the TCGA data portal. The RPM data were log2-transformed after adding one pseudocount. To identify subtype-specific miRNAs, we performed differential analyses using 2-sample *t*-tests, comparing each consensus subtype with the other subtypes.

For reverse-phase protein array analyses of protein levels, normalized measurements of 220 proteins from 215 GAC tumors were downloaded from the TCPA website (http://app1.bioinformatics.mdanderson.org/tcpa/). To identify subtype-specific protein features, we performed differential analyses using of 2-sample *t*-tests, comparing each consensus subtype with the other subtypes.

### Gastric stem cell signature and stemness scores in gastric tumors

To identify genes whose expression was significantly associated with gastric stem cells, we analyzed gene expression data of mouse pylorus cells collected from genetically engineered mice (GEMs) (GSE121803) by using the intestinal stem cell marker Lgr5 and the newly identified gastric stem cell marker Aqp5 [30]. The Lgr5-EGFP-IRES-creERT2 GEM model expresses EGFP and a CreERT2 fusion protein from the *Lgr5*promoter/enhancer elements [31]. The Lgr5-DTR-EGFP GEM model expresses EGFP and a DTR fusion protein from the *Lgr5*promoter/enhancer elements [32]. Lgr5-positive cells from GEM models were isolated by EGFP fluorescence-activated cell sorting (FACS), and Aqp5-positive cells from mouse pylorus tissue were collected by using FACS with anti-Aqp5 antibodies from wild-type mice. Gene expression data were generated by using the Affymetrix Mouse Gene 2.0 ST Array platform. A 2-step selection process was used to select the gastric stem cell signature. First, genes whose expression was associated with Lgr5 expression (Lgr5-high vs. Lgr5-low) in pylorus cells from each GEM model were selected as the Lrg5-genes for each model (*P* < 0.05, by Student *t*-test), generating 2 gene lists (2038 genes for Lgr5-EGFP-IRES-creERT2 mice and 3624 genes for Lgr5-DTR-EGFP mice). Likewise, genes whose expression was associated with Aqp5 expression (Aqp5-high vs. Aqp5-low) in pylorus cells from wild-type mice were selected as Aqp5-genes (*P* < 0.05, by Student *t*-test, 4564 genes). Second, genes whose expression significantly differed between the low and high groups in both GEM models and wild-type mice were selected as a gastric stem cell signature (356 genes). We used a Bayesian compound covariate predictor (BCCP) model as described previously to generate the probability (i.e., a stemness score) that a particular primary tumor would have a gastric stem cell gene expression signature [22, 33–35]. The BCCP was applied to standardized and combined data sets from mouse tissues (training set) and patients’ primary tumors (test set).

### Immune-response signatures of gastric tumors

To identify genes whose expression was associated with sensitivity or resistance to host immunity, we analyzed gene expression data of xenografted mouse gastric tumors established from 2 mouse gastric cancer cell lines, YTN2 and YTN16 [36]. YTN2 mouse gastric cancer tumors spontaneously regress in xenograft-bearing mice in a Cd8 T cell–dependent manner. Unless host Cd8 T cells are depleted by administration of anti-Cd8 antibodies, YTN2 tumors regress 3 weeks after transplantation in syngeneic mice. YTN16 cells spontaneously form tumors without depletion of Cd8 T cells. Gene expression data were generated from untreated mouse tumors harvested at 1 to 3 weeks after transplantation by using the HiSeq X Ten or NovaSeq 6000 platforms from Illumina. Normalized gene expression data were obtained from the Gene Expression Omnibus database (GSE146027) and analyzed to find genes whose expression was significantly different between the 2 mouse tumors, yielding 931 genes (*P* < 0.01 by 2-sample *t*-test and twofold difference) comprising the Cd8 T cell-reactive signature (CD8TRS) in gastric cancer. The BCCP algorithm was applied to standardized and combined data sets from mouse tumors (training set) and patients’ primary tumors (test set) to generate CD8TRS scores as described in the section on the generation of stemness scores.

To identify genes whose expression was associated with response to anti-PD-L1 antibodies (atezolizumab), we analyzed gene expression data of pretreated gastric tumors from the PERFECT trial, in which gastric cancer patients were treated with atezolizumab after conventional chemoradiotherapy in the neoadjuvant setting (NCT03087864) [37]. A pathological complete response (pCR) was observed in 30% of patients. Gene expression data were generated from pretreatment biopsies of 29 patients by using the HiSeq4000 platform from Illumina (GSE165252). Analysis revealed 422 genes whose expression was significantly associated with pCR after treatment (*P* < 0.05 by 2-sample t-test and 0.5-fold difference); these genes were considered as an anti-PD-L1 response signature. The BCCP algorithm was applied to standardized and combined data sets from the PERFECT trial’s tumors (training set) and primary tumors (test sets) to generate anti-PD-L1 response scores.

### ARTIST cohort for assessing efficacy of adjuvant chemoradiation in the consensus subtype

The ARTIST study was previously described [38, 39]. Briefly, 458 patients with radically resected (R0) nonmetastatic gastric cancer were enrolled and randomized to either adjuvant XP (capecitabine plus cisplatin) or XPRT (XP for 2 cycles followed by radiotherapy concurrently with capecitabine, followed by 2 additional XP cycles). The planned treatment was completed in 172 (75.4%) patients in the XP arm and 188 (81.7%) patients in the XPRT arm. Of the patients who completed treatment, tissues for NanoString nCounter experiments were available from 158 (XP) and 173 (XPRT) patients.

### Generation and analysis of gene expression data with NanoString nCounter platform

A customized panel for NanoString nCounter experiments (120 genes and 7 housekeeping reference genes) was compiled based on the gene list in the GPICS120 prediction model and stably expressed reference genes in gastric cancer tissues. Reference genes included *ACTB*, *CLTC*, *GAPDH*, *GUSB*, *HPRT1*, *RPL29*, and *TBP*. Custom codesets of 127 genes were designed according to NanoString’s standard protocol. RNA was isolated from 10 µM-thick formalin-fixed, paraffin-embedded tissue samples mounted on slides using the Roche High Pure RNA Paraffin Kit according to the manufacturer’s protocol. Concentration and fragmentation of purified RNA were assessed by using an Invitrogen qubit fluorometer and an Agilent bioanalyzer at the Advanced Technology Genomics Core at The University of Texas MD Anderson Cancer Center. Hybridization of RNA to NanoString probes was carried out according to the manufacturer’s protocol. After incubating at 65 °C for hybridization for 20 to 22 h, the reaction products were loaded to a GEN2 Prep Station for washing and immobilizing signals to a cartridge. The data were collected by Digital Analyzer (NanoString Technologies). Gene expression was processed and normalized by using the CodeSet content normalization method, which uses housekeeping genes for normalization (nSolver v 4.0 software from NanoString Technologies). The GPICS120 algorithm was applied to normalized gene expression data to stratify patients in the ARTIST cohort into consensus subtypes.

### Gene expression data from GC PDX models

Gastric cancer PDX tumors were established by Crown Bioscience as previously described [40, 41]. mRNA expression data from PDX tumors were generated on the Illumina HiSeq2500 platform. For bioinformatic analysis of transcriptome sequencing data, raw RNA sequencing data were first cleaned up by removing contamination reads that preferentially mapped to the mouse genome (UCSC MM10). Clean reads were mapped to reference genes (ENSEMBL GRCh37.66) by Bowtie, and gene expression was calculated by MMSEQ. The GPICS120 predictor was applied to gene expression data from PDX models to stratify them into the 6 consensus subtypes.

### Radiation treatment of gastric cancer cells

AGS, MKN1, SNU668, and Hs746T cells were kindly supplied by Dr. Jae-Ho Cheong (Yonsei University, Seoul, Republic of Korea). NUGC3 cells were supplied by Dr. Misun Won (Korea Research Institute of Bioscience and Biotechnology, Daejeon, Republic of Korea). All cells were grown and maintained in RPMI-1640 medium (Hyclone, Logan, UT, USA) supplemented with 10% heat-inactivated bovine serum (Hyclone) (56 °C for 30 min) and 1% penicillin–streptomycin (Corning Inc., Corning, NY, USA). Cells were maintained at 37 °C in a humidified incubator containing 5% CO_2_. Short tandem repeat DNA profiling was performed to authenticate cell lines used in the experiments.

To determine the radiation sensitivity of gastric cancer cells, colony-counting assays were conducted. Cells were first trypsinized, diluted, and enumerated; next, they were seeded onto 6-well plates in triplicate. After 24 h of incubation, the cells were exposed to different doses of X-ray radiation (0, 2, 4, 6, and 8 Gy) using an XStrahl RS225 cabinet at room temperature with 195 kV/15 mA X-rays, producing a dose rate of 1.6 Gy per minute. Irradiated and untreated control cells were subsequently cultured for 8–21 days. The surviving cell–derived colonies were stained with crystal violet solution (0.5% crystal violet in 50% methanol) and counted.

Atorvastatin (S5715) and Fer-1 (S7243) were purchased from Selleckchem (Houston, TX, USA). Simvastatin (S6196) was purchased from Sigma (St. Louis, MO, USA). Cells were treated with atorvastatin, simvastatin (Hs746T: 10 nM; SNU668:100 nM), or fer-1 (50 nM) 12 h after seeding. After 24 h of incubation, the cells were irradiated and cultured for 9–24 days. To determine whether fer-1 could abrogate statin-induced radiosensitization, cells were treated with 50 nM fer-1 24 h after irradiation. Irradiated and drug-treated cells were cultured, stained, and counted as described above.

## Results

### Meta-analysis of genomic subtypes of primary GAC

For discovery and validation of consensus genomic subtypes (CGSs) of GAC, we collected 10 GAC data sets (*N* = 2527) and divided the data into 2 groups: a discovery/training set (Korea, Samsung, TCGA, Shanxi, Ruijin, and Singapore1 cohorts, *n* = 1427) and a validation set (ACRG, Korea National Cancer Center [KNCC], Yonsei Medical Center, and Singapore2 cohorts, *n* = 1100) (Supplementary Table S1). GAC tumors in the discovery set were stratified according to the 8 genomic subtyping algorithms (Supplementary Table S2) that were independently developed and validated in previous studies [6–9, 11, 17–19]. Supplementary Figure. S1 summarizes the strategy and workflows of our analyses. In order to comprehensively integrate the subtype information from 8 classification methods and identify consensus subtypes, we used a previously established method, cluster-of-clusters assignment (COCA) [24–27], which takes as input the binary vectors that represent each subtype of the 8 classifications and reclusters the tumors according to those vectors as described in method. COCA of binary vectors revealed 6 CGSs of GAC (Fig. 1A). While the delta area plots indicated that 5 or 6 was the optimal number of subtypes, tracking plots showed that one subtype had only one sample when k = 5 (Fig. 1B,C). Therefore, we selected k = 6 as the optimal number of subtypes.

Not surprisingly, some of the 6 CGSs were subsets of previously recognized genomic subtypes. For example, the MD Anderson mesenchymal phenotype (MP) subtype was highly similar to CGS1. However, the MD Anderson epithelial phenotype (EP) subtype was split among CGS2, CGS3, CGS4, CGS5, and CGS6 (Supplementary Fig. S2A). Likewise, the genomic diffuse (GIDF) subtype was split among CGS1 and CGS4, and the genomic intestinal (GINT) subtype was split among CGS2, CGS3, and CGS5 (Supplementary Fig. S2B). In addition, the genomically stable (GS), microsatellite instability (MSI), and Epstein Barr virus (EBV) subtypes of TCGA4 classification were similar to CGS1, CGS5, and CGS6, respectively, while the chromosome instability (CIN) subtype was split among CGS2, CGS3, and CGS4 (Supplementary Fig. S2C). Taken together, these relationships suggest that the COCA approach not only rediscovered subtypes similar to previously recognized subtypes (CGS1, CGS5, and CGS6) but also discovered novel subtypes that were not recognized by previous studies (CGS2, CGS3, and CGS4). Overall, new analysis increased our confidence in these subtypes because of the largest sample size analyzed to date. Further confirming the concordance of the 6 consensus subtypes with previous classifications, the 6 consensus subtypes were significantly associated with patient prognosis (*P* = 1.5 × 10^–5^, log-rank test; Fig. 1D).

### Validation of 6 CGSs

**Fig. 2:**
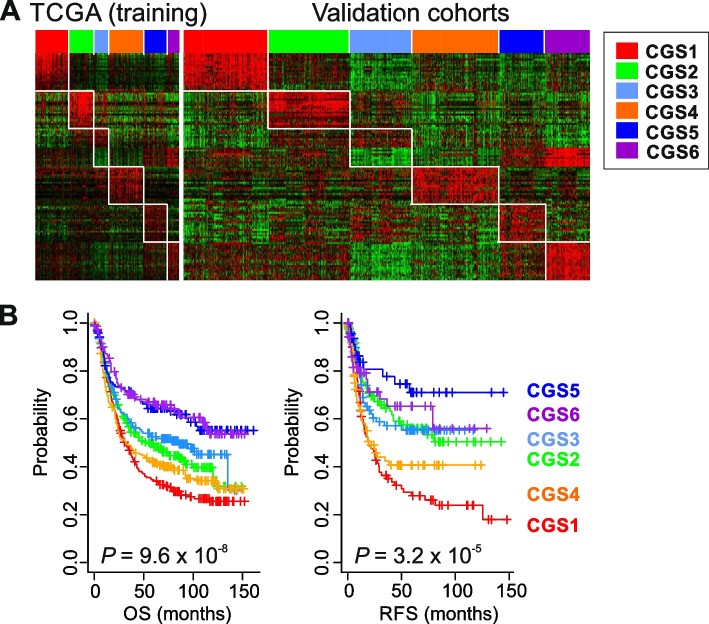
Validation of 6 consensus subtypes of gastric adenocarcinoma in validation cohorts. **A** Expression patterns of the prediction signatures for 6 consensus subtypes of GAC in training and validation cohorts. **B** Prognostic significance of consensus subtypes in validation cohorts (*n* = 1100). OS, overall survival; RFS, recurrence-free survival

Because the TCGA study provided the most comprehensive genomic profiling among the included studies, its data were selected for development of our prediction model. By applying multiple statistical tests to the gene expression data from the TCGA cohort, we selected 20 genes per subtype as a subtype-specific gene expression signature (Fig. 2A, left; Supplementary Fig. S3A). To validate the 6 subtypes and their clinical significance in independent cohort of GAC patients, we constructed the “GAC predictor of integrated consensus subtype with 120 genes” (GPICS120) by using a previously established algorithm [42, 43]. For this validation, gene expression and clinical data from the validation set (*n* = 1100) were used (Supplementary Table S1). When patients in the validation cohort were classified according to GPICS120 (Fig. 2A, right; Supplementary Fig. S3B), the 6 consensus subtypes were all significantly associated with overall survival (OS) rate (*P* = 9.6 × 10^–8^, by the log-rank test) (Fig. 2B). As in the discovery set, CGS1 and CGS4 were associated with the worst OS rates. In contrast, CGS5 and CGS6 were associated with the best OS rates. Patients in CGS2 and CGS3 had OS rates that were worse than those of CGS5 and CGS6 but better than CGS1 and CGS4. Recurrence-free survival rates of the CGSs were highly similar to OS rates (*P* = 3.2 × 10^–5^).

### Genomic, epigenomic, and proteomic characteristics of the CGSs

**Fig. 3:**
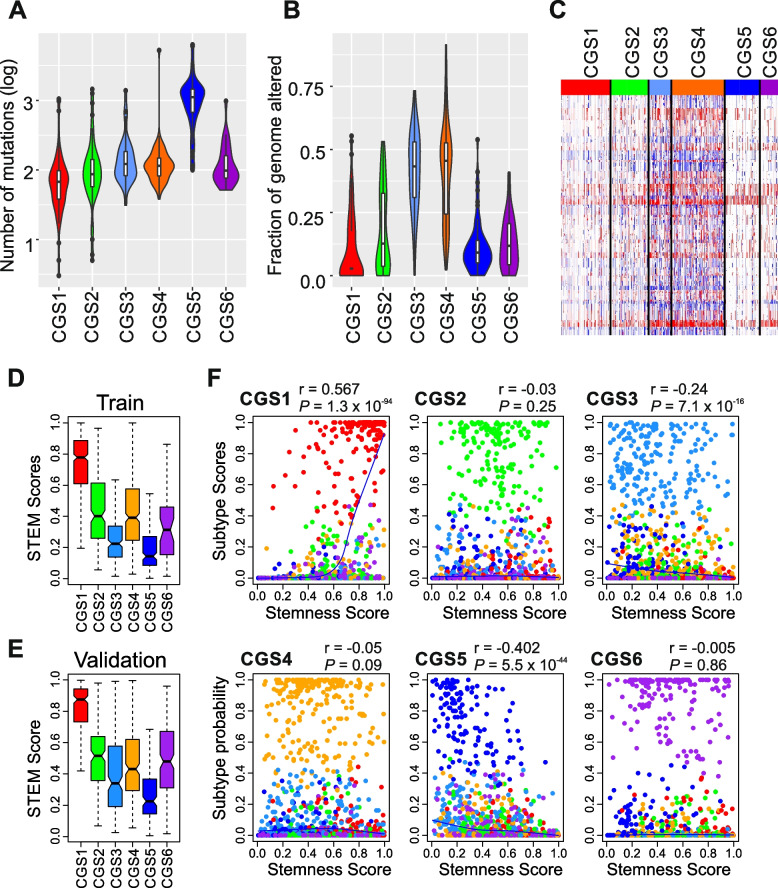
Genomic alterations and stemness scores associated with consensus subtypes. **A** Violin and box plots of the number of nonsynonymous mutations in the consensus subtypes (*n* = 338). CGS5 showed a significantly higher mutation burden than other subtypes (*P* < 0.001 in all comparisons by Student *t*-test). Within each violin, the horizontal distance between the left and right curved boundaries represents the distribution of mutations in each subtype. In the box plots, the boundaries of each box indicate the 25th to 75th percentile, and the black line within the box marks the mean. Whiskers above and below the box indicate the 10th and 90th percentiles. **B** Fraction of the genome altered by copy number gain and loss, estimated by gistic2 analysis for each tumor (*n* = 382). CGS3 and CGS4 had significantly higher proportions of copy number alterations than the other subtypes (*P* < 0.001 in all comparisons by Student *t*-test). **C** Heat map of copy number alterations of the 22 autosomes (*n* = 382). Red and blue indicate gain and loss of chromosome copy number, respectively. **D**, **E** Distribution of stemness (STEM) scores in the consensus subtypes in the training (d) and validation (e) cohorts. The boundaries of each box indicate the 25th to 75th percentile, and the black line within the box marks the mean. Whiskers above and below the box indicate the 10th and 90th percentiles. **F** Subtype probabilities from GPICS120 in the validation cohort plotted against their stemness scores. The significance is estimated by Pearson correlation coefficient

We next assessed the association of genomic and epigenomic characteristics with the 6 CGSs in the TCGA cohort to gain additional insight into each subtype’s biology. Mutation burden was significantly higher in CGS5 than in all other subtypes (*P* < 0.001 in all comparisons, Fig. 3A). Copy number alterations also significantly differed among the subtypes, with CGS3 and CGS4 having the most (Fig. 3B,C). Both of these findings suggest that differences in genomic alterations are well reflected in the consensus subtypes.

We next sought to identify mutations significantly associated with the subtypes (Supplementary Fig. S4). Owing to its high mutation burden, CGS5 had the highest number of associated mutated genes, including *ARID1A*, *XYLT2*, *RPL22*, and *PGM5* (Supplementary Fig. S5). More interestingly, the mutation rate of 2 tyrosine kinases, *ERBB3* and *ERBB4,* was significantly higher in CGS5 than in the other subtypes (Supplementary Fig. S6). *TP53* was the most frequently mutated gene, with relatively high mutation rates in all subtypes, but *TP53* mutation rate was highest in CGS3. CGS1 was most associated with *CDH1* mutations, and CGS3 was most associated with *CDH11* mutations. Interestingly, *PIK3CA* mutations were highly enriched in CGS6, suggesting that the phosphatidylinositol 3-kinase (PI3K) pathway is highly activated in CGS6.

Furthermore, an analysis of TCGA data revealed subtype-specific methylation patterns (Supplementary Fig. S7). Most strikingly, CGS6 was the subtype most strongly associated with hypermethylation, indicating a potential connection between PI3K pathway activation and hypermethylation in GAC. CGS5 was also associated with hypermethylation, albeit to a lesser degree. Interestingly, CGS1 displayed both hypermethylation and hypomethylation. However, the CGS2, CGS3, and CGS4 subtypes did not show any specific methylation pattern, suggesting that epigenetic alterations play limited roles in the development of these subtypes.

Analysis of microRNA (miRNA) expression data showed that CGS1 had the most dramatic alterations in miRNA expression (Supplementary Fig. S8). Of 286 subtype-specific miRNAs, 75% (*n* = 217) were CGS1-specific. While the vast majority of CSG1-specific miRNAs were downregulated, miRNAs miR-99a-5p, miR-99a-3p, miR-100-5p, miR-199-5p, miR-199-3p, and miR-133a-3p were overexpressed in CGS1. Interestingly, most miRNAs that were specific to the other consensus subtypes were upregulated. CGS3-specific miRNAs included miR-192-5p, miR-192-3p, miR-194-3p, and miR-194-5p. The hypermethylated subtype CGS6 was characterized by overexpression of miR-31-3p and miR-31-5p, indicating a potential connection of these miRNAs to epigenetic regulation.

Reverse-phase protein array analysis revealed CGS1, CGS5, and CGS6 to have highly distinctive proteomic characteristics (Supplementary Fig. S9). Interestingly, the CGS1 subtype was characterized by high expression of MYH11 and collagen VI and low expression of E-cadherin and α-catenin, which play essential roles in cell-to-cell interaction of epithelial cells [44], suggesting that CGS1 tumors undergo EMT that might account for the poor clinical outcomes associated with this subtype. CGS5 was characterized by high expression of claudin 7, ASNS, and 4E-BP. Immune-related signaling proteins such as STAT5, Syk, and Lck were highly expressed in CGS6, suggesting that this subtype’s high EBV load might trigger massive activation of immune cells.

In good agreement with the high likelihood of EMT in CGS1, hematoxylin and eosin staining analysis of TCGA tumors revealed CGS1 to have the highest stromal fraction and lowest tumor fraction in the tumor mass (Supplementary Fig. S10), suggesting that EMT may trigger infiltration of stromal cells into the tumor mass. mRNA expression-based estimations of tumor purity (ESTIMATE [45]) yielded consistent results.

### Stem-cell features of the subtypes

To determine the stem-cell characteristics of each GAC subtype, we generated stemness scores of GAC tumors by applying a gastric stem-cell gene expression signature, which was extracted by analyzing gene expression data from mouse gastric stem cells, to gene expression data from GAC tumors (Supplementary Fig. S11) [30]. Interestingly, the subtype with the poorest prognosis, CGS1, had the highest stemness score, whereas the subtype with the best prognosis, CGS5, had the lowest stemness score in both the training and validation cohorts (Fig. 3D,E). Consistently, a correlation analysis of the stemness score with GPICS120 probability for each subtype showed that the CGS1 subtype was significantly positively correlated with the stemness score (*r* = 0.567, *P* = 1.3 × 10^–94^), whereas the CGS5 subtype was significantly but negatively correlated with the stemness score (*r* =  − 0.402, *P* = 5.5 × 10^–44^, Fig. 3F). In agreement with this, the stemness score was significantly correlated with the TCGA recurrence risk score that was previously developed and validated for predicting recurrence after treatment (Supplementary Fig. S12) [6, 11], further supporting significant association of a stemness phenotype with poor prognosis.

### Clinical implications of the consensus subtypes for immunotherapy

**Fig. 4:**
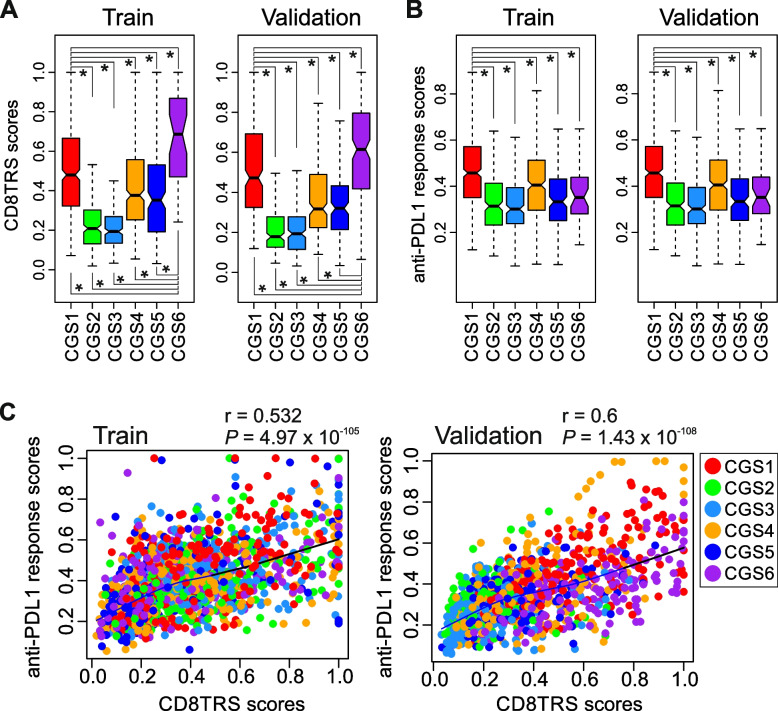
Association of consensus subtypes with treatment benefit. **A**, **B** Box plots of CD8 T-cell immunity scores (CD8TRS) (a) and anti-PD-L1 response scores (b) in consensus subtypes. The boundaries of each box indicate the 25th to 75th percentile, and the black line within the box marks the mean. Whiskers above and below the box indicate the 10th and 90th percentiles. *P < 0.001 by Student *t*-test. **C** Scatter plots of CD8 T-cell immunity scores and anti-PD-L1 response scores in training and validation cohorts. Colors indicate consensus subtypes as indicated. Black lines indicate loess regression

Immunotherapy with antibodies that the block inhibitory immune checkpoint regulators has yielded encouraging results in GAC patients [46–48]. However, not all patients benefit from immunotherapy [4, 49, 50]. Because this unequal benefit might be caused by intrinsic sensitivity or resistance of cancer cells to host immune activity, we assessed these features in each subtype by using gene expression signatures from mouse gastric tumors that are sensitive or resistant to CD8 T cell-dependent anticancer immunity (CD8 T-cell reactive signature [CD8TRS] scores (Supplementary Fig. S13) [36]. CGS6, with the highest fraction of EBV-positive tumors, had the highest CD8TRS score, i.e., the highest sensitivity to CD8 T cell–dependent anticancer immunity (Fig. 4A). Interestingly, CGS1, with the poorest prognosis and highest stemness, had the second highest CD8TRS score, suggesting that tumors in this subtype might be sensitive to CD8 T cell–dependent anticancer immunity or related immunotherapy. CGS3 and CGS4, which had high genomic instability, had lower CD8TRS scores. Interestingly, CSG1 and CGS6, which had high CD8TRS scores, had very low genomic instability. In agreement with this observation, CD8TRS score was significantly negatively correlated with genomic instability (Supplementary Fig. S14A).

To further validate these findings, we next applied a gene expression signature significantly associated with response to treatment with a neoadjuvant anti-PD-L1 antibody (atezolizumab) in patients with gastroesophageal cancer to generate anti-PD-L1 response scores (Supplementary Fig. S15) [37]. Consistent with the CD8TRS scoring results, CGS3 and CGS4 subtypes had the low potential response to anti-PD-L1 or anit-PD-1 agents, while CGS1 had the highest potential response (Fig. 4B). However, contrary to its CD8TRS score, the CGS6 subtype had only a moderate potential response to anti-PD-L1 treatment. Nonetheless, overall, CD8TRS score was significantly correlated with anti-PD-L1 response score (Fig. 4C), supporting the conclusion that these 2 independently generated immunity signatures reflect anticancer immunity in GACs. As was the CD8TRS score, anti-PD-L1 response score was significantly but negatively correlated with genomic instability (Supplementary Fig. S14B, strongly supporting a potential mechanistic connection between chromosomal instability and host immune response. Taken together, these results indicate that the consensus subtypes are strongly associated with potential response to immunotherapy.

### Clinical implications of the consensus subtypes for chemoradiation therapy

**Fig. 5:**
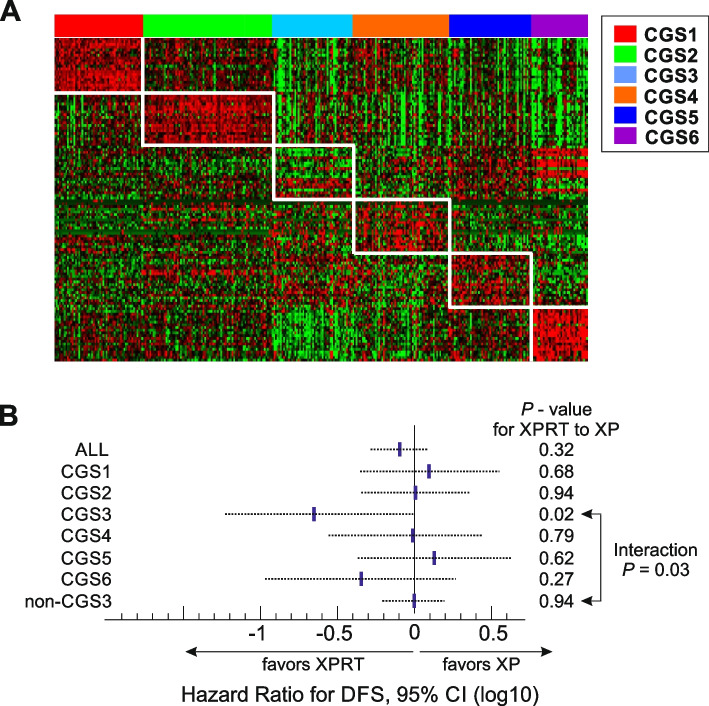
High sensitivity of CGS3 subtype to radiation therapy. **A** Expression patterns of GPICS120 genes in gastric tumor tissues from ARTIST cohort (*n* = 331). Tumors were stratified according to GPICS120. Subtype-specific gene expression patterns were well conserved in the tumors from the ARTIST cohort. **B** Forest plots showing hazard ratio (HR) for recurrence for adjuvant XPRT treatment over XP treatment in consensus subtypes of GAC tumors from patients in the ARTIST clinical trial. A Cox proportional hazard regression model was used to analyze the interaction between DFS of patients with CGS3 tumors and the rest of patients (non-CGS3 tumors and XPRT treatment. The dotted line represents the 95% confidence interval (CI) of HRs. XP, capecitabine + cisplatin; XPRT, XP + radiotherapy

Although adjuvant chemotherapy has become the standard treatment for GAC after surgery [51], the benefit of adjuvant chemoradiation therapy is not clearly defined [38, 39, 52]. Thus, we sought to determine whether the consensus subtypes were associated with clinical benefit from adjuvant chemoradiation therapy. To do this, we used the NanoString nCounter platform to generate gene expression data of GACs from patients in the ARTIST trial who were treated with adjuvant capecitabine and cisplatin (XP) or XPRT (XP followed by radiotherapy) and followed for disease-free survival (DFS) as the primary endpoint [38, 39]. Of the registered patients, we included only those who completed treatment and had available tissues in the analysis (*n* = 331, Supplementary Fig. S16A). Similar to the original report, addition of radiotherapy to XP did not significantly reduce recurrence after surgery in the included patients (Supplementary Fig. S16B. Application of GPICS120 to the gene expression data of the 331 ARTIST tumors stratified them into the 6 consensus subtypes (55 for CGS1, 84 for CGS2, 46 for CGS3, 60 for CGS4, 51 for CGS5, and 35 for CGS6, Fig. 5A). Most importantly, subtype CGS3 showed a significant benefit from XPRT treatment compared to XP treatment (Fig. 5B). For CGS3, the hazard ratio (HR) for recurrence in patients receiving XPRT treatment compared to XP treatment was 0.22 (95% confidence interval [CI], 0.058–0.81, *P* = 0.02, Fig. 5B). However, no significant benefit from XPRT treatment was observed in the rest of the subtypes, suggesting that the benefit of the addition of radiotherapy might be attributable to differences in the underlying biology among subtypes.

### Potential therapeutic targets in consensus subtypes

Because previous studies showed that trastuzumab, which targets HER2 (*ERBB2*), is the most effective treatment for GAC with genomic copy number amplification or high expression of HER2 [53], we assessed copy number alterations of *HER2* in the consensus subtypes. Interestingly, CGS3 had the highest amplification of *HER2* among the 6 subtypes (Supplementary Fig. S17). In agreement with this, mRNA expression of *HER2* was also highest in CGS3, and its expression and copy number were significantly positively correlated More interestingly, methylation of the *HER2* promoter was lowest in CGS3, and HER2 expression and promoter methylation were significantly but negatively correlated, suggesting that HER2 expression in CGS3 is increased by both genetic and epigenetic alterations.

Interestingly, similar to *HER2*, expression of *SALL4* was positively correlated with copy number but negatively correlated with promoter methylation (Supplementary Fig. S18), indicating that its expression in GAC is also regulated by both genetic and epigenetic alterations. However, unlike *HER2*, *SALL4* expression was highest in CGS4. Next, because a recent study showed that amplification is the major activation mechanism of *KRAS*in GAC cancer and that KRAS amplification is significantly associated with resistance to MAPK blockade [54], we assessed copy number alterations of *KRAS* by subtype. *KRAS* amplification was highest in CGS4 and was significantly associated with *KRAS* mRNA expression (*r* = 0.81, *P* = 1.2 × 10^–90^, Supplementary Fig. S19). However, unlike that of *HER2* and *SALL4*, *KRAS* promoter methylation was not significantly associated with mRNA expression, indicating that *KRAS* activation in GAC is mainly driven by copy number alteration.

One of the genes most significantly overexpressed in the poorest-prognosis CGS1 subtype was *IGF1* (Supplementary Fig. S20), a ligand for IGF1R that is a known potential therapeutic target for many cancers [55, 56]. mRNA expression of *IGF1* was negatively correlated with *IGF1* promoter methylation (*r* =  − 0.459, *P* = 2.1 × 10^–21^) but not with copy number alteration, indicating that activation of *IGF1* in GAC is largely mediated by epigenetic mechanisms.

### Consensus subtypes in preclinical GAC models

We next applied GPICS120 to the gene expression data of 114 GAC patient-derived xenograft (PDX) tumors. Of these tumors, 6, 14, 26, 31, 30, and 7 were classified as subtypes CGS1, CGS2, CGS3, CGS4, CGS5, and CGS6, respectively (Supplementary Fig. S21A). Importantly, the expression patterns of the GPICS120 genes were well conserved in PDX tissues, suggesting that PDX tumors recapitulate the molecular and biological characteristics of primary GAC well. Interestingly, most PDX tumors appeared to be stable, as the passage number of PDX models differed only slightly among subtypes, except for CGS1, which was much lower (Supplementary Fig. S21B).

**Fig. 6:**
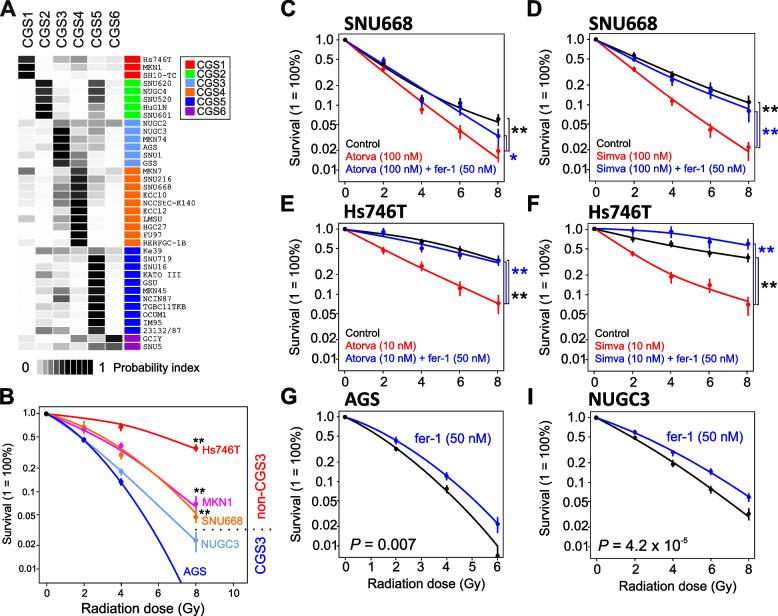
Sensitivity of GAC cell lines to radiation treatment. **A** Consensus subtypes in GAC cell lines (*n* = 37 in CCLE data set). GAC cell lines were stratified according to nearest shrunken centroids probability from the GPICS120 predictor. **B** Log-linear plot demonstrating the relative sensitivity of the 5 GAC cell lines (AGS, NUGC3, SNU668, MKN1, and Hs746T) to ionizing radiation over the range of 0 to 8 Gy as determined by a colony-counting assay. Each point represents the mean of 5 replicates. **P < 0.001 for AGS or NUGC3 vs. other cells. **C**-**I** Inhibition of ferroptosis induces resistance to ionizing radiation and abrogates statin-mediated radiosensitization. Colony-counting assays showed that ferrostatin-1 (fer-1), an inhibitor of ferroptosis, abrogates statin-mediated radiosensitization in radiation-resistant SNU668 and Hs746T gastric cancer cells (**C**-**F**). Furthermore, fer-1 makes radiation-sensitive AGS and NUGS3 gastric cancer cells resistant to ionizing radiation (**G**, **I**). Seeded cells were exposed to ionizing radiation over the range of 0 to 8 Gy as indicated. *P*-values indicate the significance of the differences at the 8-Gy dose in each experiment. Means (± SEM) of at least 3 experiments are shown. Atorva, atorvastatin; Simva, simvastatin. *P < 0.01, **P < 0.001

Because established cancer cell lines are the most practical experimental models, we applied GPICS120 to gene expression data of GAC cell lines from the Cancer Cell Line Encyclopedia (CCLE) data set and stratified 37 cell lines according to GPICS120 (Fig. 6A) [57]. We next examined the associations of the consensus subtypes with sensitivity to ionizing radiation in these cell lines. In agreement with the clinical association of CGS3 subtype with radiation sensitivity in the ARTIST cohort, 2 cell lines of the CGS3 subtype, AGS and NUGC3, were more sensitive to radiation than were cell lines of other subtypes (Fig. 6B), suggesting that these cell lines may recapitulate the biology of radiation sensitivity at a cellular level. To uncover the underlying biology associated with sensitivity to radiation in CGS3 tumors, we next performed network analysis with genes specific to the CGS3 subtype. Since the 20 genes specific to CGS3 in GPICS120 were too few to run network analysis, we re-selected genes that were differentially expressed in CGS3 tumors compared to non-CGS3 tumors, yielding 1188 genes. Next, we performed gene network analysis to identify potential upstream effectors or regulators of 1188 genes. Interestingly, a large fraction of predicted effectors of gene networks in CGS3 were chemical drugs (Supplementary Fig. S22). In particular, multiple statins predicted to regulates genes specific to CGS3 subtype (Supplementary Table S3, Supplementary Fig. S23), indicating that the cellular state of CGS3 tumors is similar to that of tumor cells treated with statins. This association of statins with genes specific to the radiation-sensitive CGS3 subtype suggests that statins may sensitize GAC tumors to radiation treatment.

To test this possibility, we treated non-CGS3 cell lines with statins during radiation exposure. In agreement with the predictions of the network analysis, both atorvastatin and simvastatin sensitized 2 non-CGS3 cell lines (SNU668 and Hs746T) to ionizing radiation (Supplementary Fig. S24). Since statins are known to promote ferroptosis by inhibiting synthesis of coenzyme Q10, which is the end product of the mevalonate pathway and a major agent for reducing oxidized fatty acids [58, 59], we postulated that statins sensitize GAC cells to radiation by enhancing baseline ferroptosis. Indeed, treatment with ferrostatin-1 (fer-1), an inhibitor of ferroptosis, abrogated statin-induced radiation sensitivity, strongly suggesting that radiation sensitivity is mediated by an increase of ferroptosis (Fig. 6C-F). In good agreement with this, fer-1–treated CGS3-subtype cells (AGS and NUGC3) were resistant to radiation (Fig. 6G, I), further supporting the notion that sensitivity to radiation in the CGS3 subtype is mediated by ferroptosis.

## Discussion

**Fig. 7:**
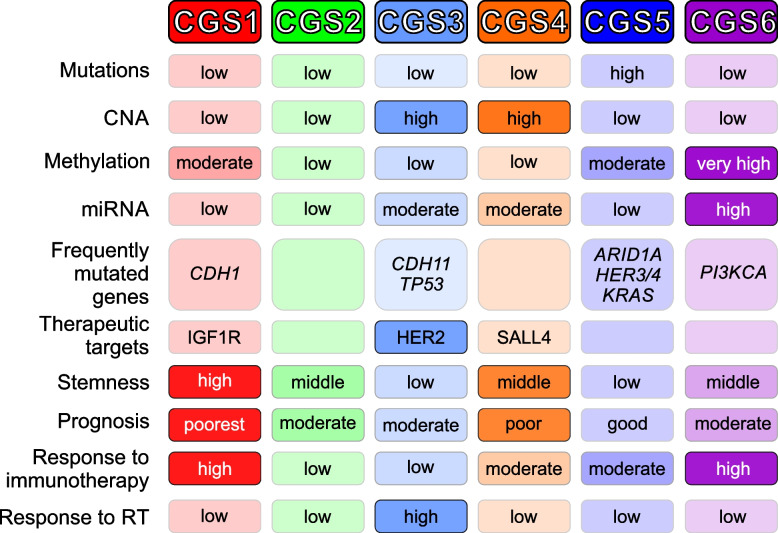
Summary of the characteristics of the 6 consensus subtypes of GAC. The 6 subtypes are well reflected by their biological and clinical characteristics

To form a consensus from independently identified molecular subtypes of gastric cancer, we integrated and reanalyzed subtype information for 8 previously discovered genomic subtypes. Our novel analysis identified 6 distinct genomic subtypes of GAC (Fig. 7). Importantly, clinical outcomes, including overall survival rates and potential responses to treatment, differed significantly among the subtypes. Our results indicate that these 6 consensus subtypes likely offer new and enhanced information regarding the underlying biology and clinical behavior of GAC.

CGS1 is characterized by the poorest prognosis, strongest stem cell features, and a high potential response to immunotherapy. While gene expression in CGS1 tumors was the most similar among the subtypes to that from mouse gastric stem cells, it is currently unknown if this high similarity reflects the origin of cancer cells or the high fraction of cancer stem cells in the tumor mass. Because acquisition of EMT is a common event in poor-prognosis tumors with stem-cell features, the high expression of mesenchymal homeobox transcription factor BARX1, one of CGS1-specific genes in GPICS120, suggests that BARX1 might contribute to EMT and the stem cell phenotype. In fact, previous studies showed that BARX1 is an essential factor for stomach development during organogenesis [60]. In agreement with this, the mesenchymal marker VIM was another CGS1-specific gene in GPICS120, providing further support that EMT is a major phenotype of CGS1. Importantly, CGS1 is significantly associated with CD8 T cell–-mediated eradication of cancer cells and potential response to anti-PD-L1 or anti-PD-1 treatment, although this finding needs further validation in preclinical and clinical studies.

The CGS2 subtype has gene expression patterns similar to those of normal gastric epithelial cells, as reflected by its high expression of *GKN1*, *GKN2*, *LIPF*, *MUC5AC*, and *MUC6*. Because the stemness score of the CGS2 subtype is not the lowest among the 6 subtypes, it is unlikely that differentiation status is not fully accountable for expressing normal gastric epithelial genes, but likely that CGS2 GAC cells partially maintain a normal physiologic transcriptome program even after malignant transformation. This subtype also was associated with a better prognosis than CGS1 but a worse one than CGS5. Notably, the CGS2 subtype was similar to a normal-like subtype in breast cancer that also expressed normal epithelial genes and has a mid-range prognosis [61]. Although major drivers of the CGS2-subtype have not been identified yet, the shared cellular features in 2 different tumor types indicate that maintaining a normal transcriptomic program may provide a certain advantage to cancer cells, for instance by allowing them to evade anticancer immune activity.

The CGS3 subtype is characterized by low expression of the cytotoxic T-cell marker CD8A, immune cytolytic genes such as *GZMB* and *GZMH*, and chemokine ligands such as CCL5, CXCL9, and CXCL10, suggesting that anticancer immunity is suppressed in this subtype. In fact, the CGS3 subtype has the lowest CD8TRS and anti-PD-L1 response scores among the 6 subtypes. Although its mutation burden is relatively low, its overall genomic instability is very high, as reflected in its high number of copy number alterations. Because previous studies indicated that high genomic instability is significantly associated with poor response to anti-CTLA-4 immunotherapy [62, 63], high genomic instability might account for the low immune activity in the CGS3 subtype. In contrast to the low immune activity in this subtype, CGS3 tumors have high expression of HER2 because of both genomic copy number amplification and promoter demethylation, indicating that tumors of the CGS3 subtype might be more sensitive to targeting of HER2 with trastuzumab [53]. Importantly, the CGS3 subtype is significantly associated with a benefit from the addition of radiation therapy to adjuvant chemotherapy, suggesting that CGS3-subtype cancer cells might be more sensitive to radiation than other subtypes. The high sensitivity of CGS3 GAC cells to radiation was further demonstrated in a cell culture model. Analysis of genomic data suggested that the CGS3 subtype may have a high basal level of ferroptosis, as reflected by the enrichment of a gene set regulated by statins that induces ferroptosis in cancer cells. In good agreement with these predictions, statin treatment sensitized radiation-resistant CGS3 GAC cells to ionizing radiation. Therefore, a novel therapeutic approach using a statin drug in addition to chemoradiation would warrant further investigation in preclinical models.

Like CGS3, CGS4 has high genomic instability. However, in CGS4 tumors, *KRAS* is one of the most highly amplified and highly expressed. Previous observations also showed that *KRAS *is frequently activated via amplification in gastroesophageal cancer [54]. Like *HER2* in CGS3, high expression of *SALL4*in CGS4 is attributed by both gene amplification and promoter demethylation. SALL4 is a C2H2 zinc finger transcription factor that is essential for embryonic development and frequently activated in many cancers [64]. Because recent studies identified SALL4 as a neosubstrate of the molecular glue thalidomide and its derivatives [65, 66], it will be interesting to determine the efficacy of thalidomide in CGS4 tumors in future preclinical models. In addition to the *KRAS* and *SALL4*oncogenes, CGS4 tumors highly express cancer-testis antigens (CTAs) such as MAGEA3 and MAGEA12, indicating that they might be good candidates for testing CTA-mediated cancer vaccine therapy [67].

CGS5 and CGS6 are characterized by high mutation rates and genomic hypermethylation, respectively. Interestingly, while CGS5 showed the highest number of subtype-specific mutations owing to its high overall mutation burden, *PI3KCA* is most highly mutated in the CGS6 subtype, suggesting that CGS6 tumors might be more sensitive to inhibitors of the PI3K pathway. Similar to the CGS1 subtype, the CGS6 subtype has high basal level immune activity as reflected in its high CD8TRS scores.

This study had some limitations. While the CGS3 subtype–specific association with radiation sensitivity is highly interesting, it was only tested in a single prospective cohort and thus needs to be validated in independent prospective studies. Regarding the associations of the consensus subtypes with immune response, because the association of the CGS1 subtype with the immune response signature was only moderate and tested only in retrospective cohorts, it should be not be extrapolated without further validation in prospective cohorts. Regardless of these limitations, it is important to point out that CGS3 is one of 3 novel subtypes (CGS2, CGS3, and CGS4) identified by our study that were not recognized in previous studies, highlighting the importance and significance of our approach of integrating multiple genomic subtype information into the analysis.

## Conclusion

Systematic analysis of genomic and proteomic data of GAC uncovered 6 subtypes that are significantly associated with benefit of standard and experimental treatments. In particular, CGS3 subtype is more sensitive to radiation therapy owing to high basal level of ferroptosis. The newly identified subtypes likely offer new and enhanced information regarding the underlying biology and clinical behavior of GAC. In summary, we identified 6 clinically distinct GAC consensus subtypes that are each homogeneous molecularly and genomically. Furthermore, the identified genetic and epigenetic alterations associated with the 6 subtypes will provide opportunities to develop new intervention for GAC and the potential marker genes we identified are preserved in PDX models, providing a valuable resource in this context.

### Supplementary Information

**Additional file 1.**

## Data Availability

Raw and processed gene expression data are available under GSE26899, GSE2690, GSE13861, GSE66229, GSE66229, GSE29272, GSE54129, GSE29998, GSE15459, GSE14208, GSE14209, and GSE84437 at the Gene Expression Omnibus database.

## Abbreviations

GAC: Gastric adenocarcinoma
MP: Mesenchymal phenotype
EP: Epithelial phenotype
GS: Genome stable
EBV: Epstein Barr virus
CIN: Chromosome instability
TCGA: The Cancer Genome Atlas
ACRG: Asian Cancer Research Group
MSS: Microsatellite stable
EMT: Epithelial-to-mesenchymal transition
CGA: Consensus genomic subtypes
KNCC: Korea National Cancer Center
COCA: Cluster-of-clusters assignment
GDIF: Genomic diffuse
GINT: Genomic intestinal
GPICS120: GAC predictor of integrated consensus subtype with 120 genes
OS: Overall survival
*ARID1A*: *AT rich interactive domain 1A (SWI-like)*
*ASNS*: *asparagine synthetase (glutamine-hydrolyzing)*
*BARX1*: *BARX homeobox 1*
*CDH1*: *cadherin 1, type 1, E-cadherin (epithelial)*
*CDH11*: *cadherin 11, type 2, OB-cadherin (osteoblast)*
*ERBB2*: *v-erb-b2 erythroblastic leukemia viral oncogene homolog 2, neuro/glioblastoma derived oncogene homolog (avian)*
*ERBB3*: *v-erb-b2 erythroblastic leukemia viral oncogene homolog 3 (avian)*
*ERBB4*: *v-erb-a erythroblastic leukemia viral oncogene homolog 4 (avian)*
*KRAS*: *v-Ki-ras2 Kirsten rat sarcoma viral oncogene homolog*
*LCK*: *lymphocyte-specific protein tyrosine kinase*
*MYH11*: *myosin, heavy chain 11, smooth muscle*
*PGM5*: *phosphoglucomutase 5*
*PIK3CA*: *phosphoinositide-3-kinase, catalytic, alpha polypeptide*
*RPL22*: *ribosomal protein L22*
*SALL4*: *sal-like 4 (Drosophila)*
*SYK*: *spleen tyrosine kinase*
*TP53*: *tumor protein p53*
*XYLT2*: *xylosyltransferase II*
PDX: Patient-derived xenograft
XP: Capecitabine and cisplatin
XPRT: Capecitabine and cisplatin followed by radiotherapy
ARTIST: Adjuvant chemo RadioTherapy In Stomach Tumors
