# Supplementary material for: Clinically conserved genomic subtypes of gastric adenocarcinoma
Source: Mol Cancer. 2023 Sep 6;22:147. doi: 10.1186/s12943-023-01796-w (PMC10481468; doi:10.1186/s12943-023-01796-w)
Supplement: Supplementary file 1 — Additional file 1. [file 12943_2023_1796_MOESM1_ESM.pdf]

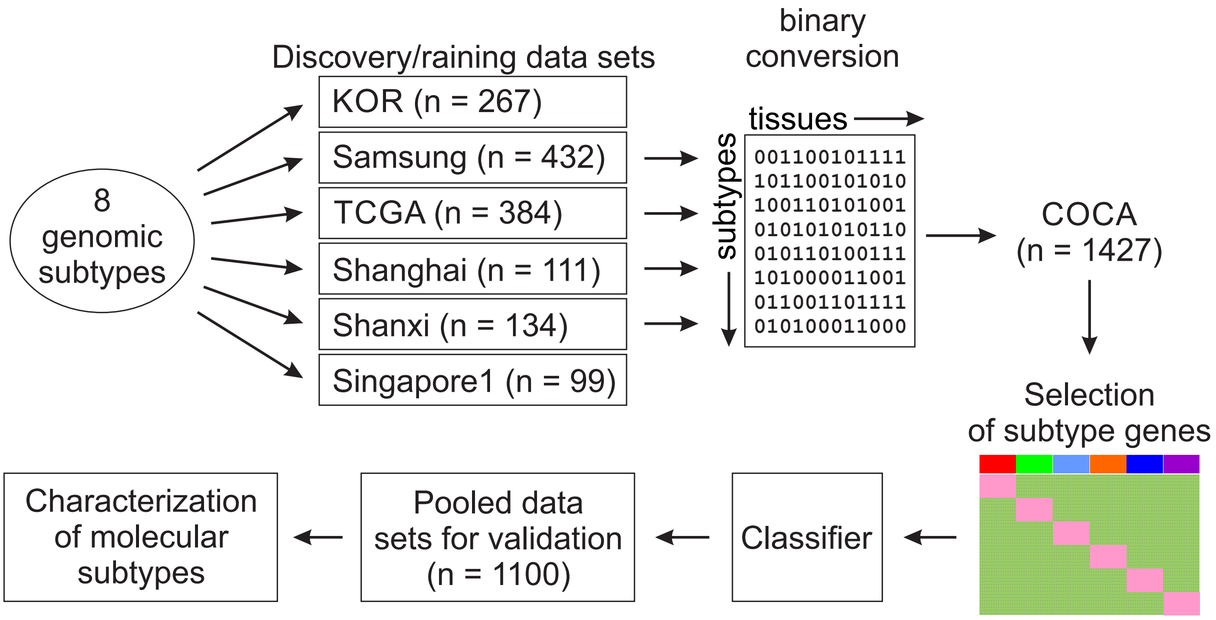

Supplementary Figure S1. Schematic diagram of the strategy for classification of the consensus subtypes.

KOR, Korea cohort; TCGA, The Cancer Genome Atlas; COCA, cluster-of-cluster assignment.

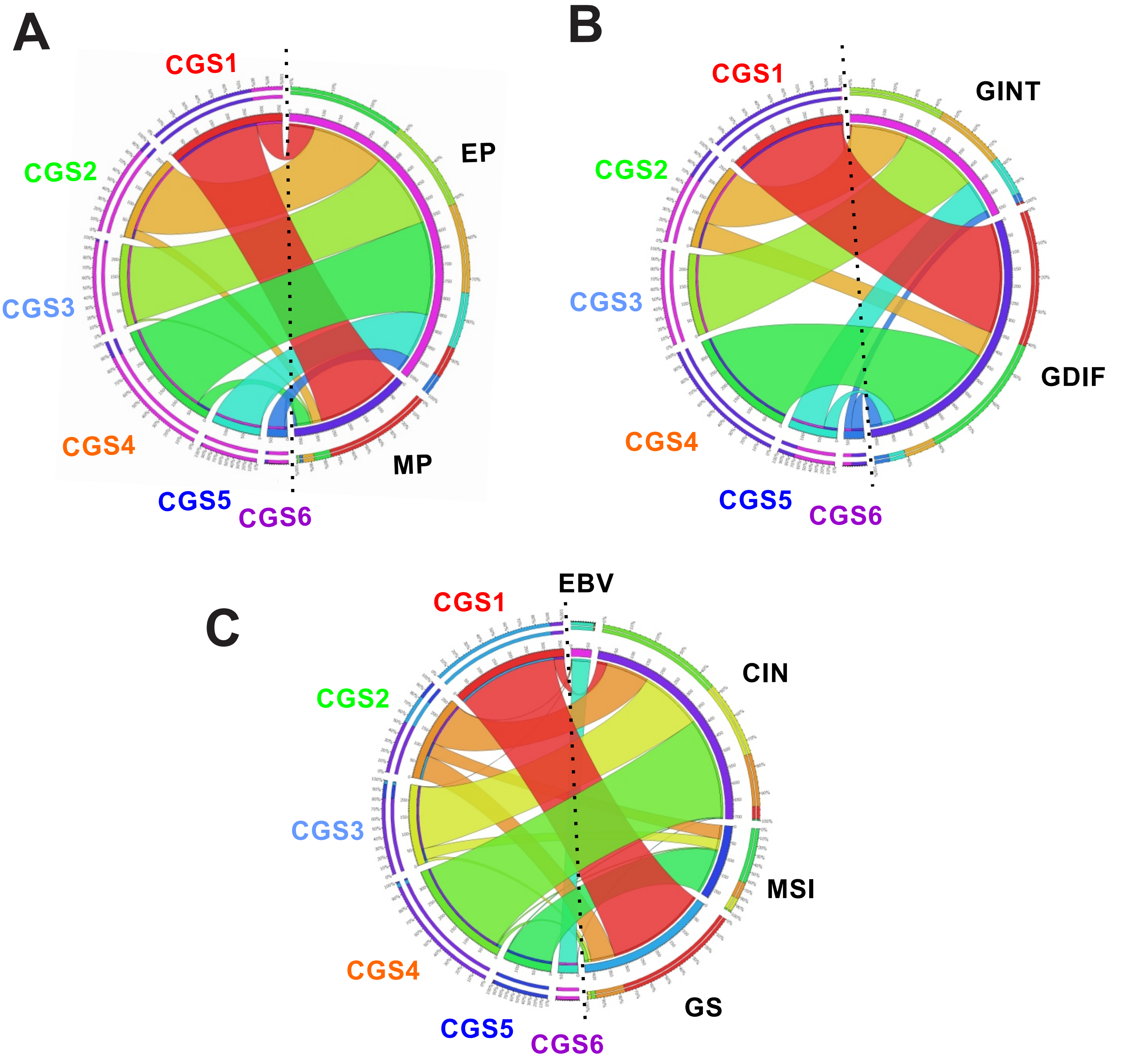

Supplementary Figure S2. Circos plots showing the relationships of TCGA4, MP, and intrinsic subtypes to the consensus genomic subtypes in the discovery/training set.

In these circular ideograms of pairwise comparisons of 2 classification systems, the ribbons indicate matched samples between 2 classification systems. Consensus subtypes are shown on the left, and matched subtypes from previous studies are displayed on the right. **A,** CGS vs. MP-EP subtype. **B,** CGS vs Intrinsic subtype. **C,** CGS vs. TCGA subtype. Graphics were built using Circos (http://circos.ca/). MP, mesenchymal phenotype; EP, epithelial phenotype; GDIF, genomic diffuse; GINT, genomic intestinal; TCGA, The Cancer Genome Atlas; GS, genome stable; MSI, microsatellite instable; CIN, chromosome instable; EBV, Epstein Barr virus.

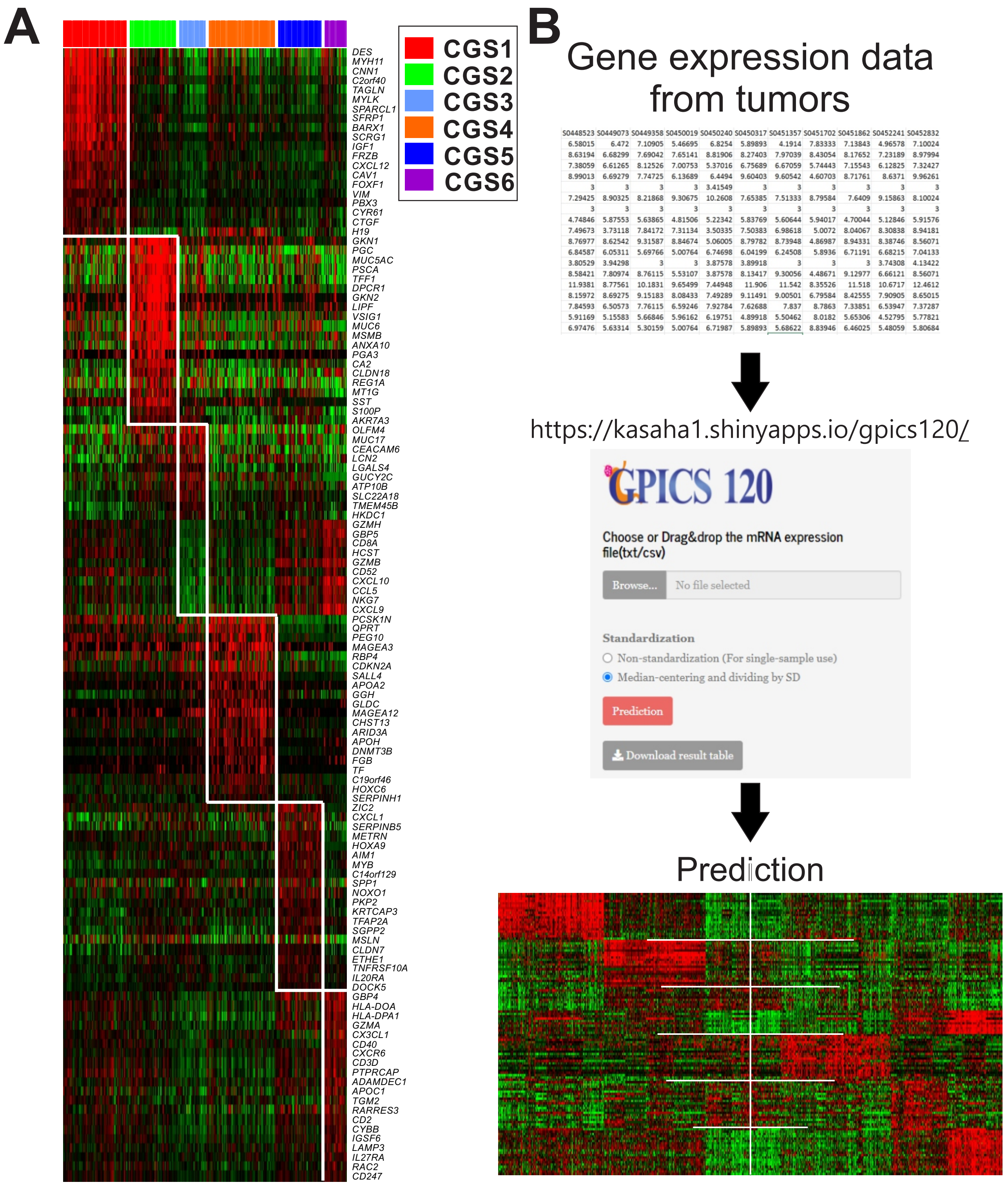

Supplementary Figure S3 GPICS120 predictor.
A, Expression patterns of prediction signatures for 6 consensus subtypes in the TCGA training cohort (n = 384 tissues). Multiple 2-sample t-tests were conducted to identify subtype-specific genes in gene expression data from this cohort. For selection of subtype CGS1 genes, 5 2-sample t-tests (CGS1 vs. CGS2, CGS1 vs, CGS3, CGS1 vs. CGS4, CGS1 vs. CGS5, and CGS1 vs. CGS6 comparisons) were carried out. Of the genes whose expression significantly differed in 4 of the 5 t-tests (P < 0.01), the top 20 genes for each subtype were selected. Expression patterns of 120 selected genes from TCGA data are presented.
B, Schematic diagram demonstrating how to use the GPICS120 web-based predictor. Expression of 120 genes from gastric cancer tumors can be directly loaded to the Shiny webserver (https://kasaha1.shinyapps.io/gpics120/). The web-based GPICS120 predictor provides outcomes of predictions, a heatmap of the query data set, and circular faction plots. Gene expression data from the validation set are provided as an example/practice data set.

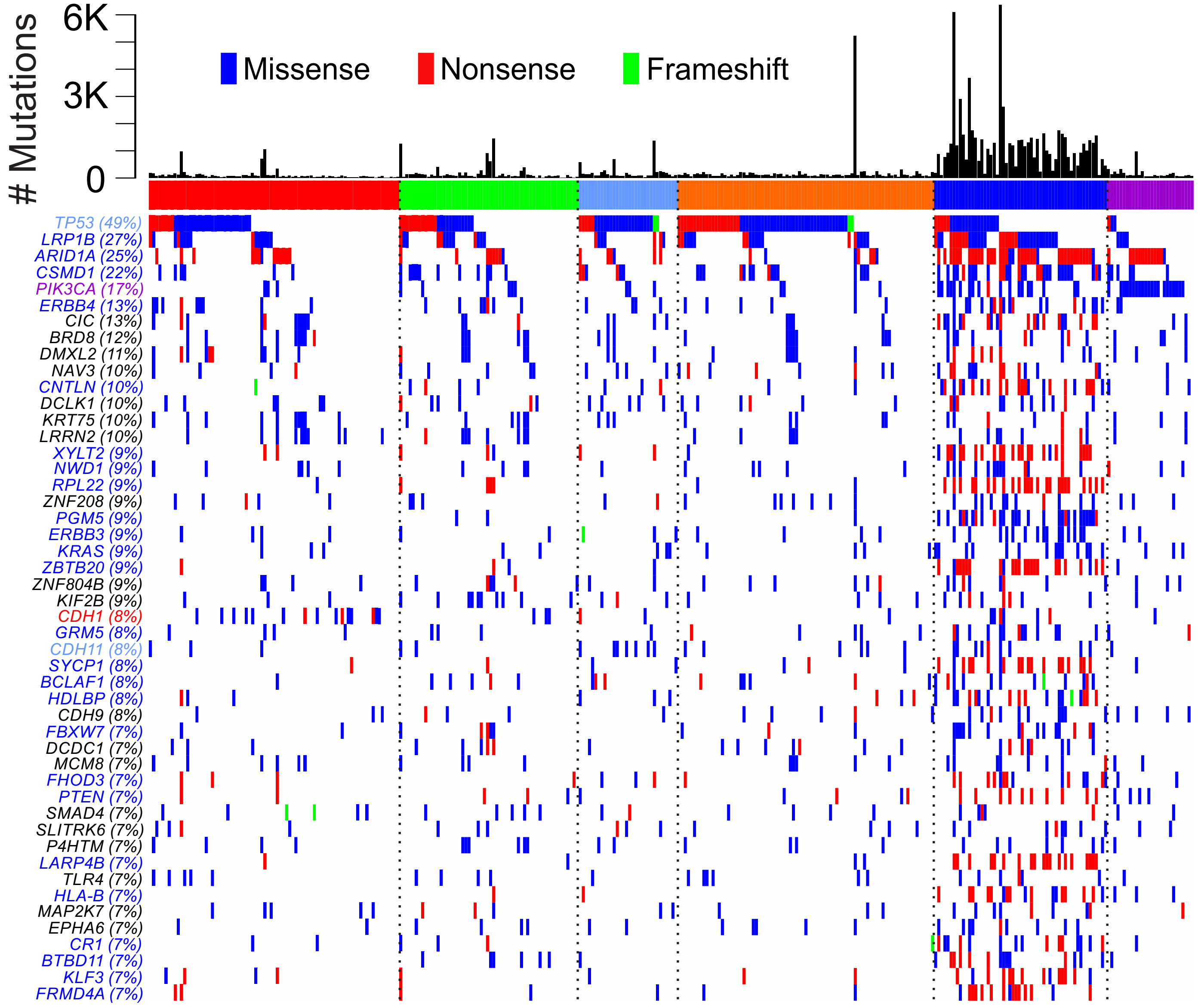

Supplementary Figure S4. Profile of somatic mutations in consensus subtypes in TCGA cohort.
The top panel shows individual tumor mutation rates; the bottom panel shows genes with statistically significant levels of mutation (MutSig suite, false discovery rate, 0.1 and >7% mutation rate). Mutation types are indicated in the legend at the top. Gene symbols in colored letters indicate genes significantly associated with the corresponding subtypes (P < 0.01).

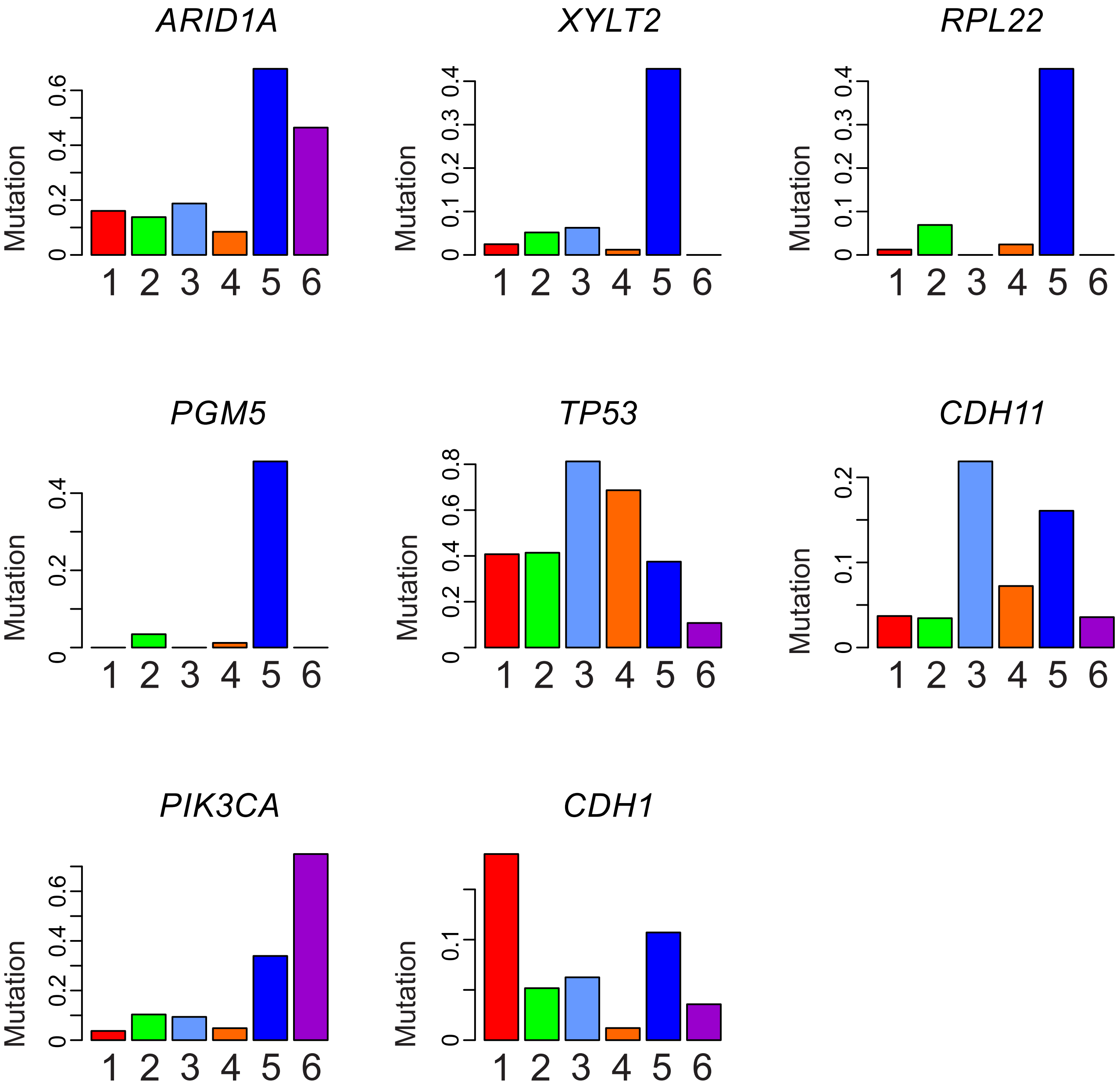

Supplementary Figure S5. Somatic mutations associated with consensus subtypes in TCGA cohort.

Mutation rates of each gene are presented as fractions within subtypes.

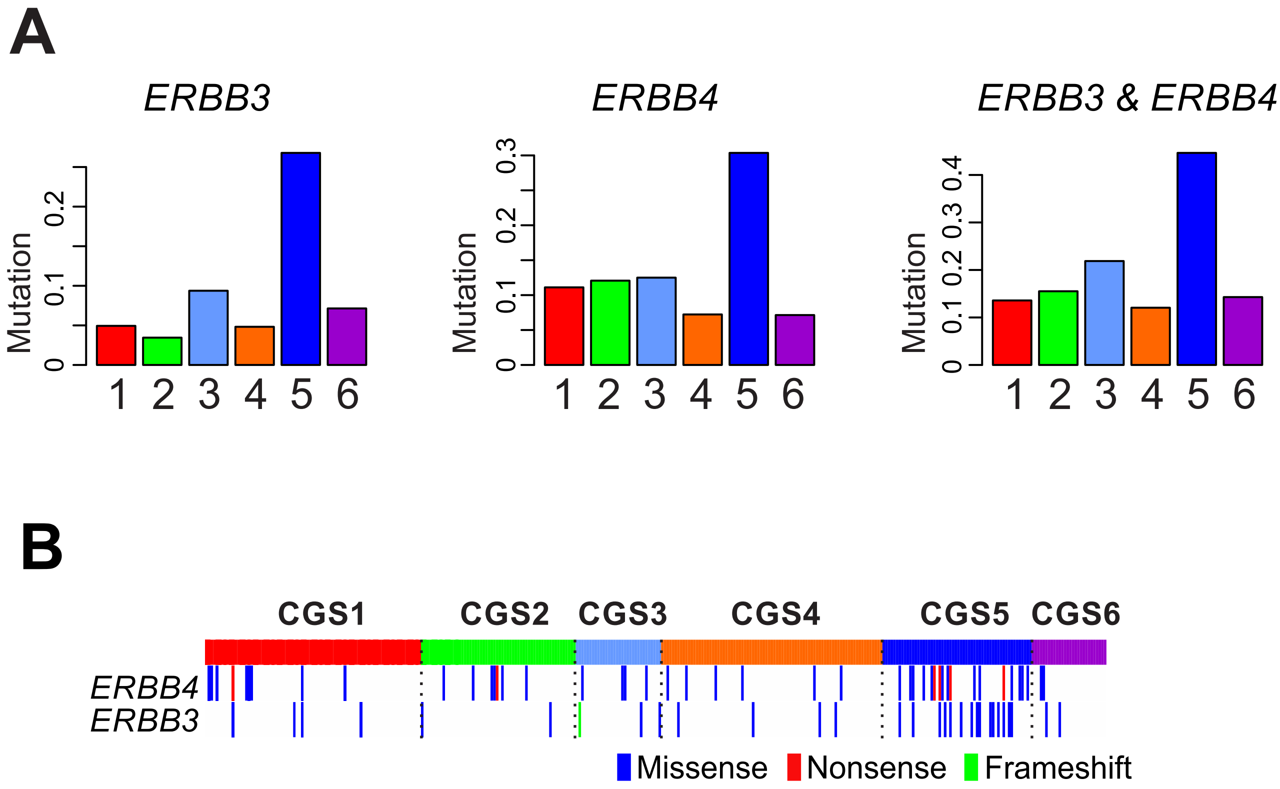

Supplementary Figure S6. Mutations of ERBB3 and ERBB4 in consensus subtypes in TCGA cohort.
A, Mutation rates of ERBB3 and ERBB4 are presented as percentages within subtypes. Mutation rates of these genes were significantly higher in CGS5 than in other subtypes ($P < 0.01$ by $\chi 2$ test). The combined mutation rate was 45% in CGS5.
B, Distribution of mutations in ERBB3 and ERBB4 in consensus subtypes.

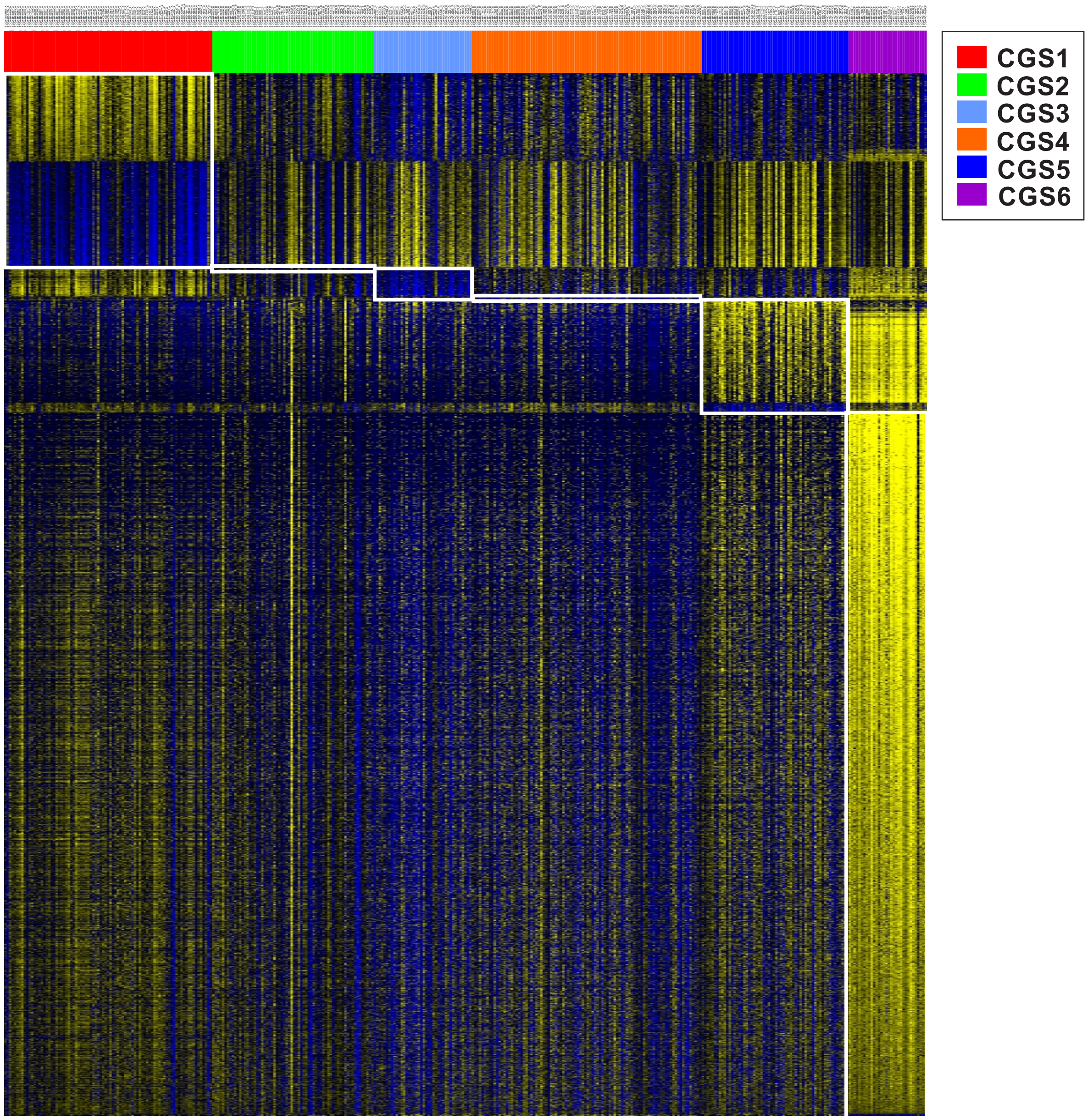

Supplementary Figure S7. Methylation patterns specific to the 6 consensus subtypes of gastric cancer in the TCGA cohort.

Multiple 2-sample t-tests were used to identify subtype-specific methylation patterns in the TCGA cohort (n = 377). For the selection of subtype CGS1, 5 2-sample t-tests (CGS1 vs. CGS2, CGS1 vs. CGS3, CGS1 vs. CSG4, CGS1 vs. CGS5, and CGS1 vs. CGS6) were carried out. Only subtypes CGS1, CGS5, and CGS6 showed significant ($P < 0.001$) alterations of DNA methylation.

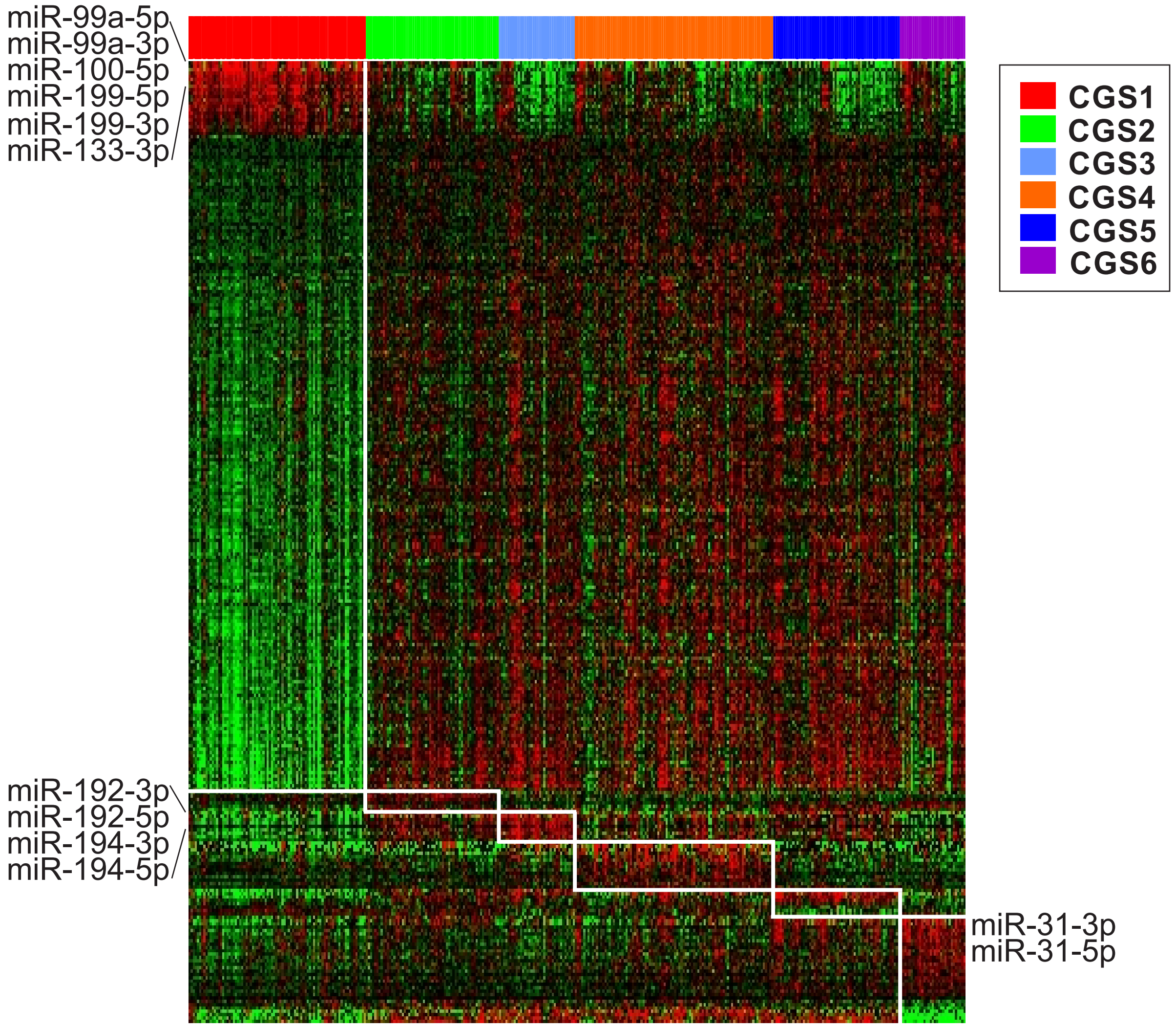

Supplementary Figure S8. miRNA expression in the consensus subtypes in the TCGA cohort.

Multiple 2-sample t-tests were carried out to identify subtype-specific miRNAs in the TCGA cohort (n = 368). For the selection of subtype CGS1, 5 2-sample t-tests (CGS1 vs. CGS2, CGS1 vs. CGS3, CGS1 vs. CSG4, CGS1 vs. CGS5, and CGS1 vs. CGS6) were carried out. A total of 286 miRNAs had subtype-specific expression (P < 0.01): 217 for CGS1, 6 for CGS2, 9 for CGS3, 14 for CGS4, 8 for CGS5, and 32 for CGS6.

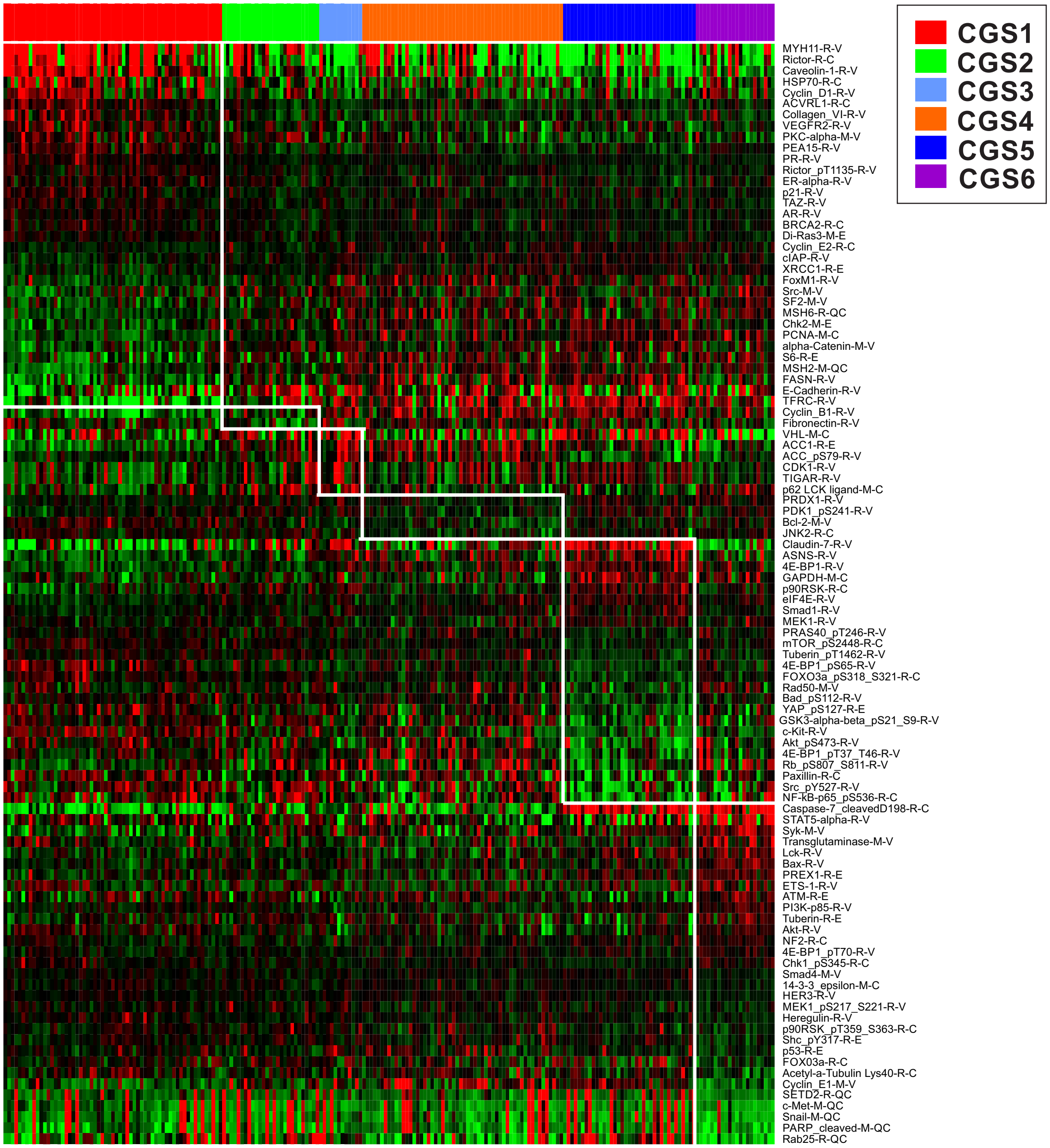

Supplementary Figure S9. Protein expression in the consensus subtypes in the TCGA cohort.

Multiple 2-sample t-tests were carried out to identify subtype-specific protein features in the TCGA cohort (n = 215). For the selection of subtype CGS1, 5 2-sample t-tests (CGS1 vs. CGS2, CGS1 vs. CGS3, CGS1 vs. CSG4, CGS1 vs. CGS5, and CGS1 vs. CGS6) were carried out. A total of 100 protein features had subtype-specific expression ($P < 0.05$): 33 for CGS1, 2 for CGS2, 6 for CGS3, 4 for CGS4, 24 for CGS5, and 31 for CGS6.

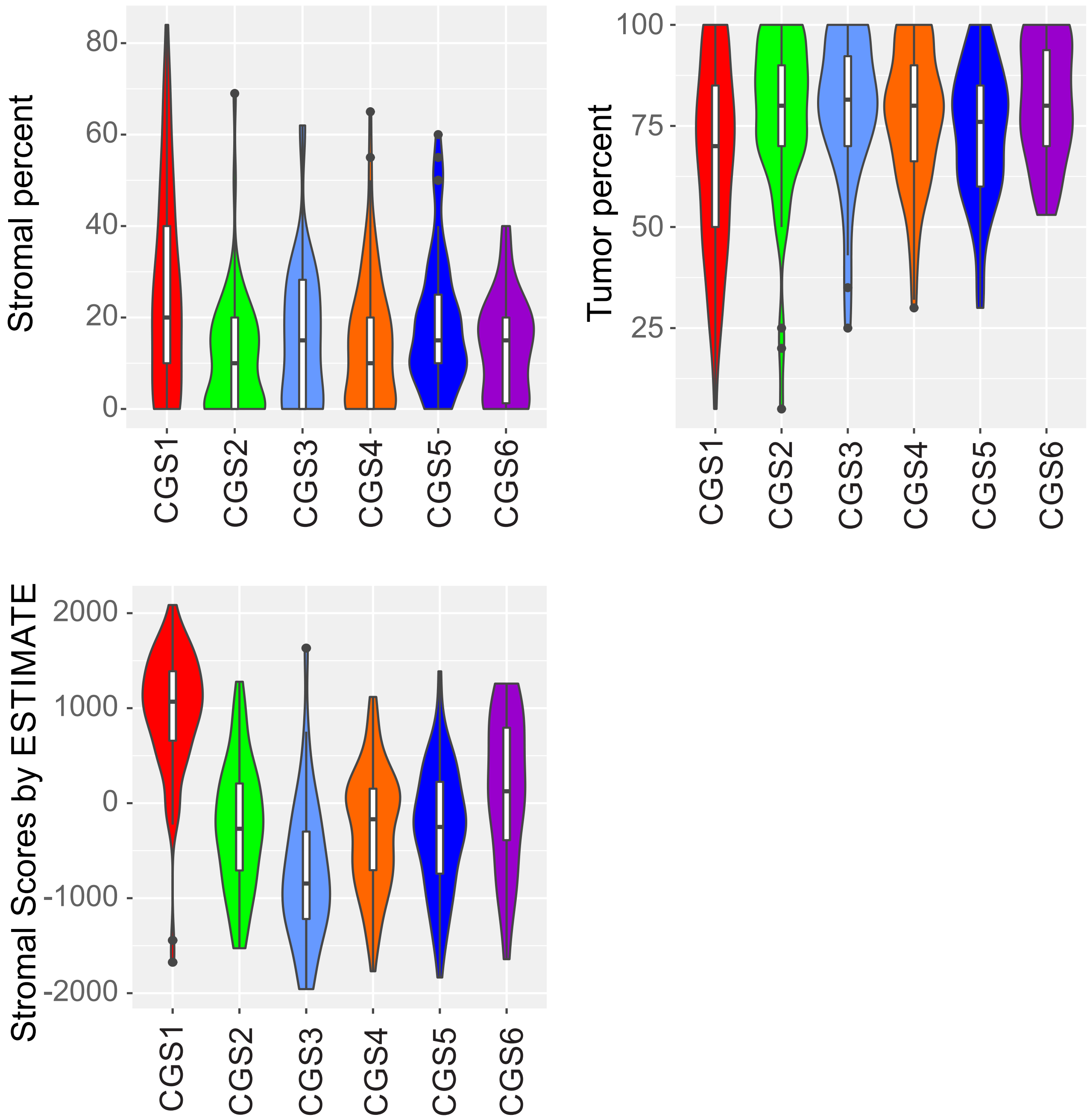

Supplementary Figure S10. Tumor purity of the consensus subtypes in the TCGA cohort.

Each tumor's purity was estimated by hematoxylin and eosin (H&E) staining and mRNA expression (ESTIMATE). Within each violin, the horizontal distance between the left and right curved boundaries represents the distribution of the percentage of stroma, tumor, or ESTIMATE scores. In the boxes, the boundaries of each box indicate the 25th to 75th percentile, and the black line within the box marks the mean. Whiskers above and below the box indicate the 10th and 90th percentiles.

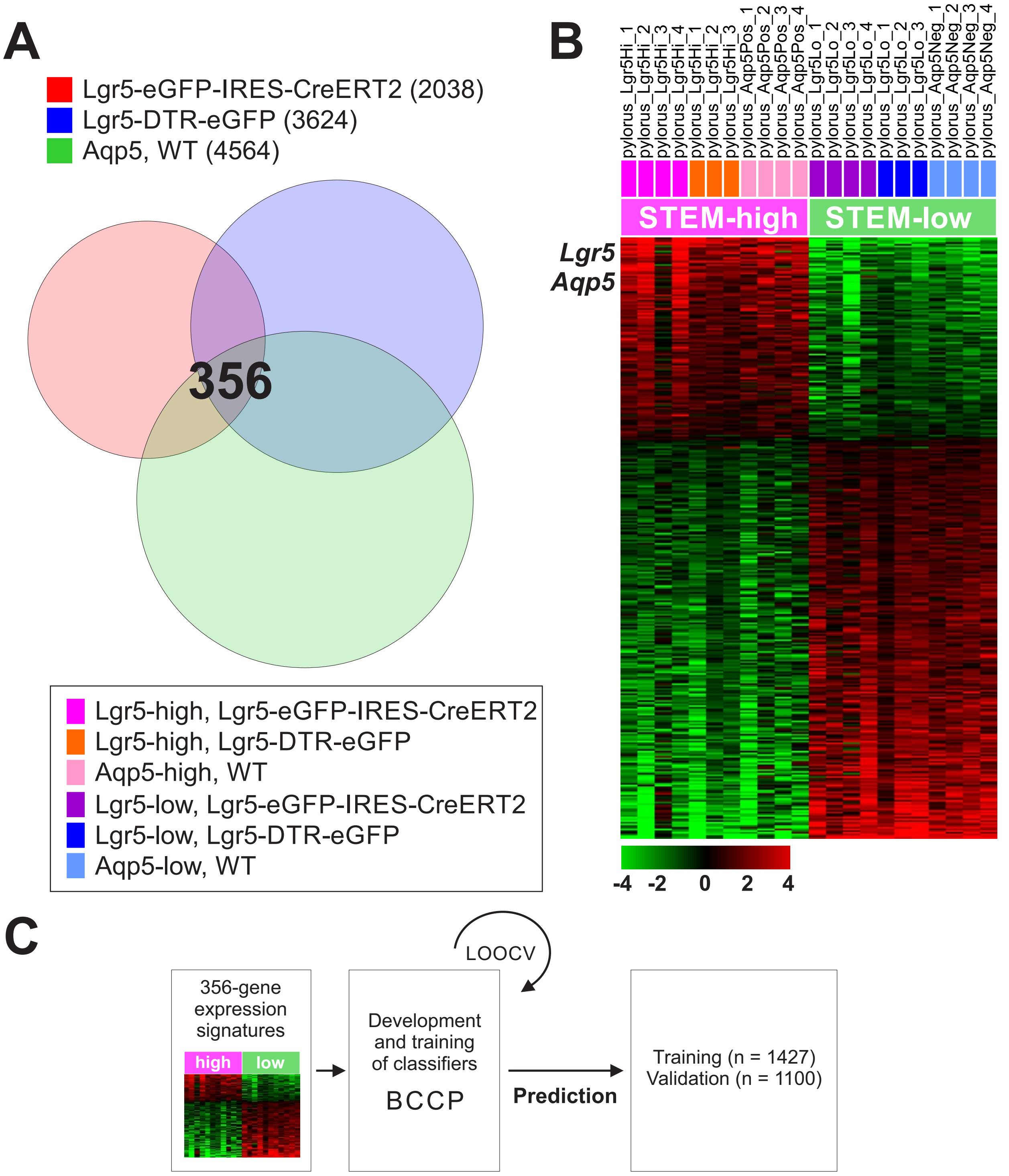

Supplementary Figure S11. Gastric stem cell signature from mice.

A, Venn diagram for selection of gastric stem cell genes from mouse pylorus cells. First, genes whose expression was associated with Lgr5 expression level (Lgr5-high vs. Lgr5-low) in pylorus cells from genetically engineered Lgr5-eGFP-IRES-CreERT2 and Lgr5-DTR-eGFP mice (GEM) were selected as Lrg5-genes. Second, genes whose expression was associated with Aqp5 expression level (Aqp5-high vs. Aqp5-low) in pylorus cells from wild-type (WT) mice were selected as Aqp5-genes. Genes whose expression was significantly different ($P < 0.05$, by Student t-test) in 2 groups of each GEM or WT mice were selected as the gastric stem cell signature (356 genes).

B, Heatmap of gastric stem cell (STEM) signature from mouse pylorus cells. Data are presented in matrix format; each row represents an individual gene, and each column represents a sample. Each cell in the matrix represents the expression level of a gene in an individual tissue sample. Red and green indicate relatively high and low expression levels, respectively, as indicated in the log2-transformed scale bar.

C, Schematic diagram of prediction model for generating stemness scores in GC tumors. BCCP, Bayesian compound covariate predictor; LOOCV, leave-one-out cross validation.

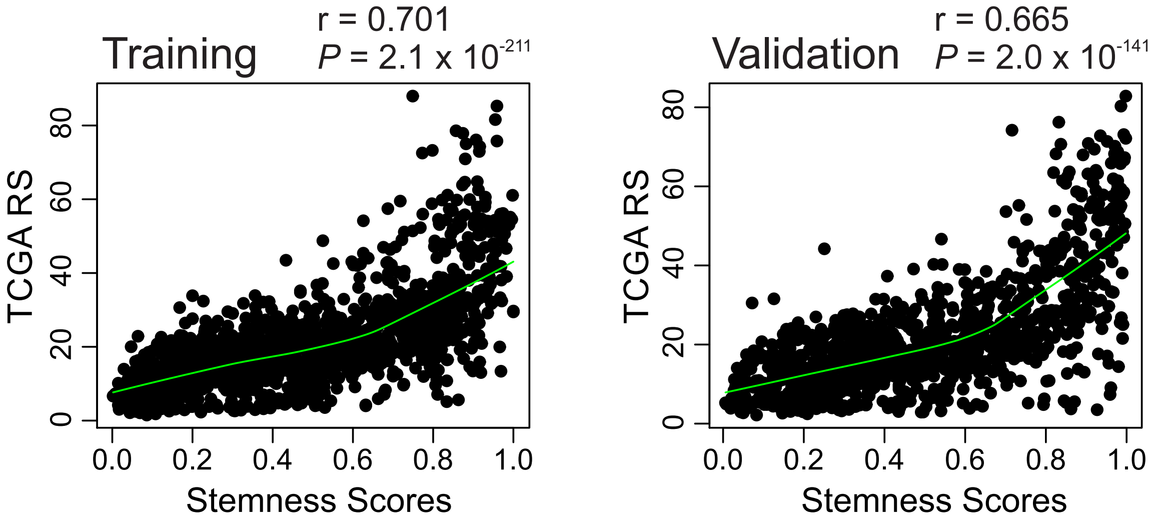

Supplementary Figure S12. Association of stemness score with recurrence risk score.

Stemness score was significantly correlated with previously developed TCGA recurrence risk scores (RS) in training and validation cohorts. Green lines indicate lowess regression. The significance is estimated by Pearson correlation coefficient.

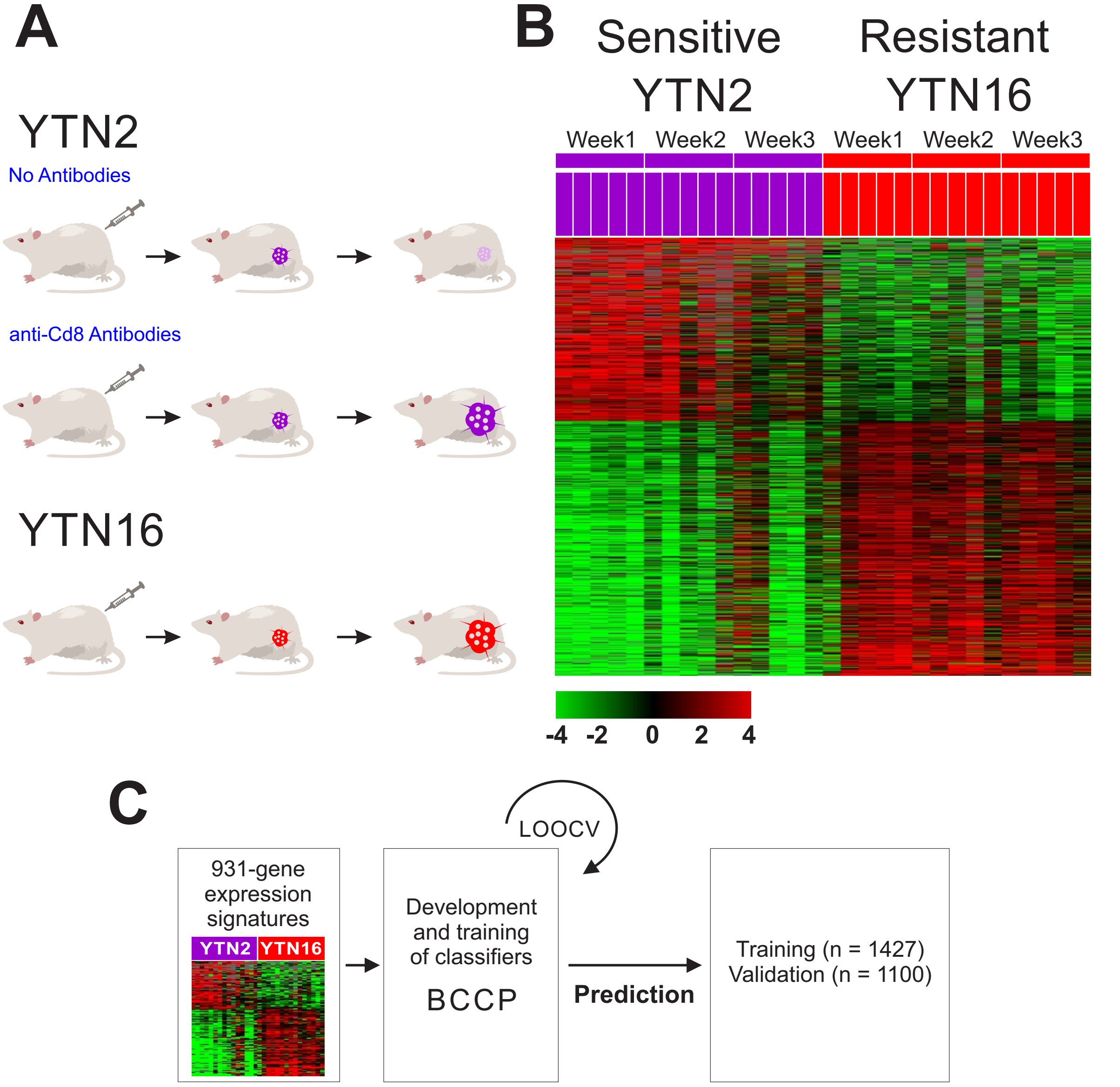

Supplementary Figure S13. CD8 T cell-reactive gene expression signature from mouse gastric tumors.

A, YTN2 mouse gastric cancer tumors spontaneously regress in xenograft-bearing mice in a Cd8 T cell-dependent manner. Depletion of Cd8 T cells by administration of anti-Cd8 antibodies to mice makes YTN2 cells form tumors in mice. YTN16 cells form tumors well without depletion of Cd8 T cells (reference 30).
B, Gene expression data were generated from untreated mouse tumors harvested at 1 week to 3 weeks after transplantation, and 931 genes whose expression was significantly different between YTN2 and YTN16 tumors were selected ($P < 0.01$ and 2-fold difference) as the Cd8 T cell-reactive signature (CD8TRS) in gastric cancer. Heatmap shows CD8TRS gene expression data at the indicated time points. Data are presented in matrix format; each row represents an individual gene, and each column represents a sample. Each cell in the matrix represents the expression level of a gene in an individual tissue sample. Red and green indicate relatively high and low expression levels, respectively, as indicated in the log2-transformed scale bar.
C, Schematic diagram of prediction model for generating CD8TRS scores in gastric cancer tumors. BCCP, Bayesian compound covariate predictor; LOOCV, leave-one-out cross validation.

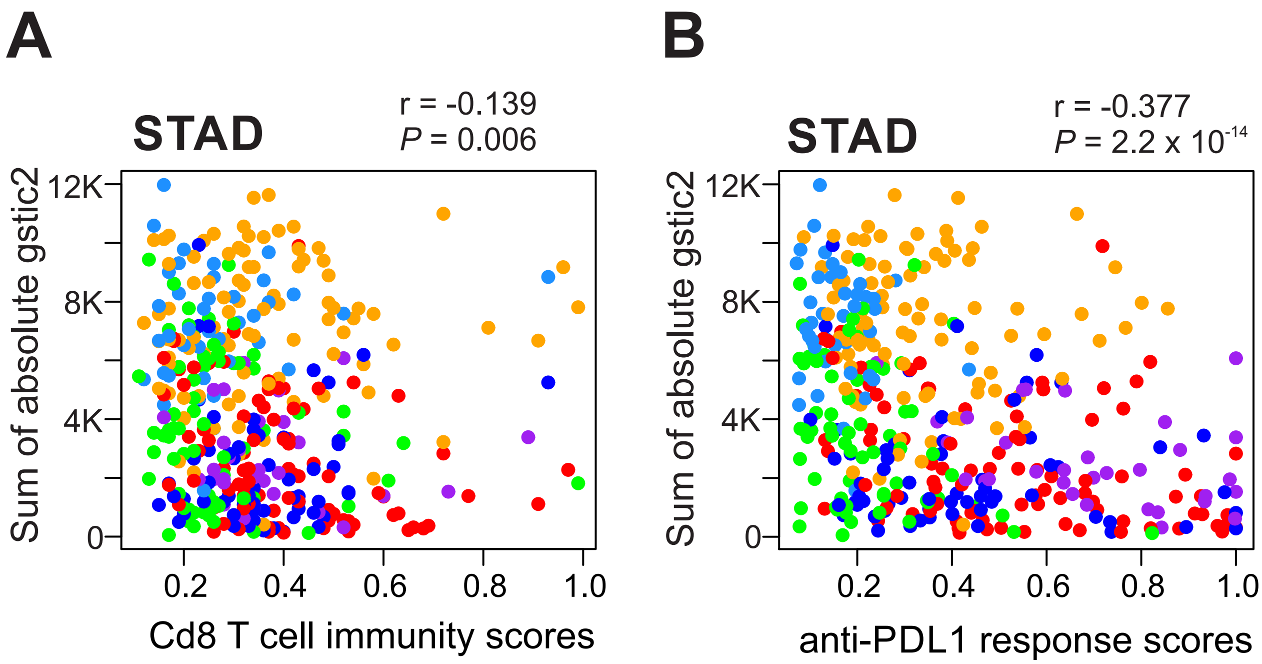

Supplementary Figure S14. Correlation of anti-cancer immunity scores with genome instability in gastric cancer. Cd8 T-cell immunity scores (**A**) and anti-PD-L1 response scores (**B**) were negatively correlated with genome instability in the TCGA gastric cancer cohort (n = 384). Genome instability is represented by summation of absolute gistic2 scores in individual tumors.

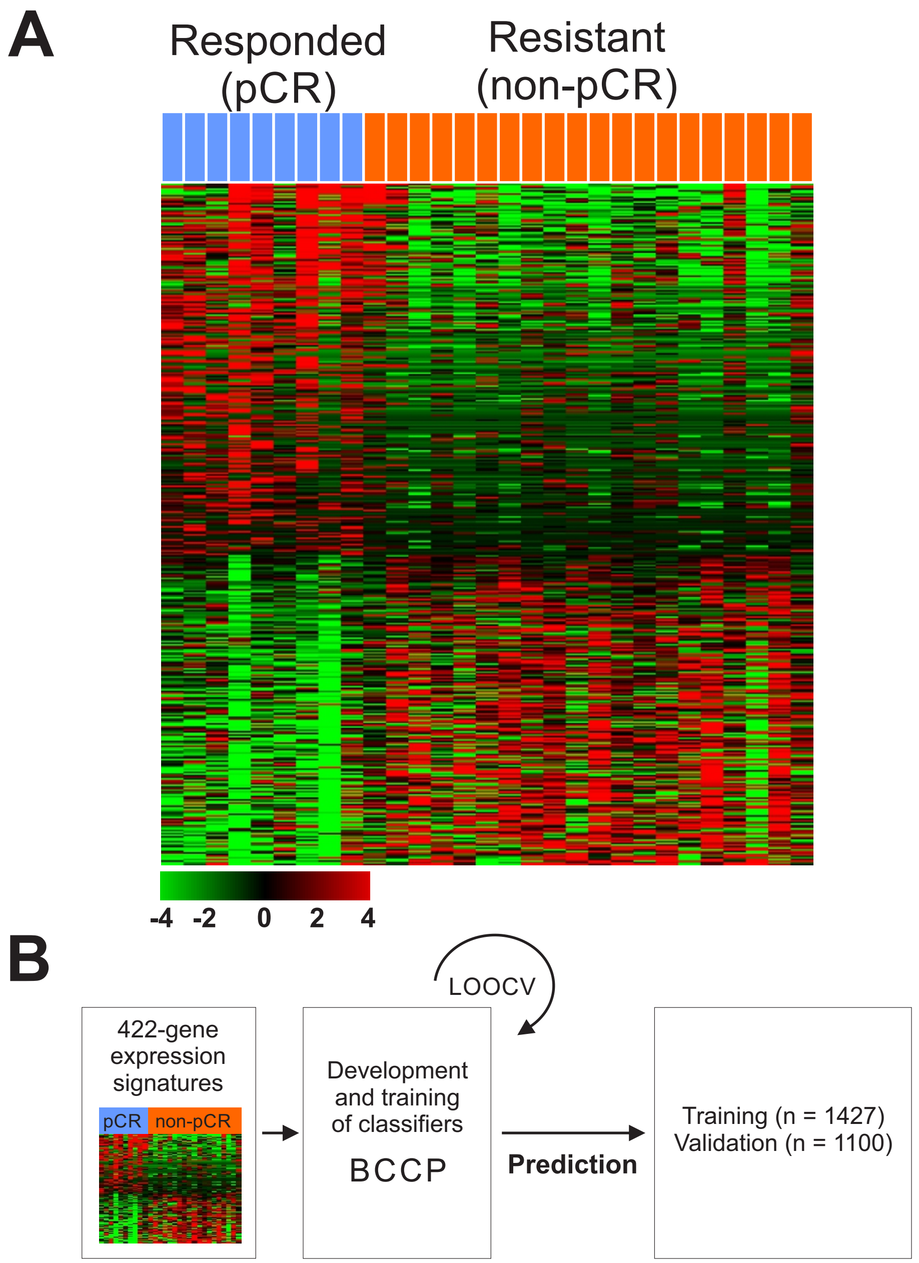

Supplementary Figure S15. Gene expression signature associated with response to anti-PD-L1 antibody (atezolizumab) treatment in gastroesophageal tumors.

A, 33 patients with resectable gastroesophageal cancer were treated with neoadjuvant chemoradiotherapy combined with intravenous atezolizumab (1200 mg) (PERFECT trial, NCT03087864, reference 31). A pathological complete response (pCR) was observed in 30% (10/33) of patients. Gene expression data were generated from pretreatment biopsy samples from 29 patients, and 422 genes whose expression was significantly associated with pCR after treatment were selected as the anti-PD-L1 response signature ($P < 0.05$ and 0.5-fold difference).
B, Schematic diagram of the prediction model for generating anti-PD-L1 response scores in GAC tumors. BCCP, Bayesian compound covariate predictor; LOOCV, leave-one-out cross validation.

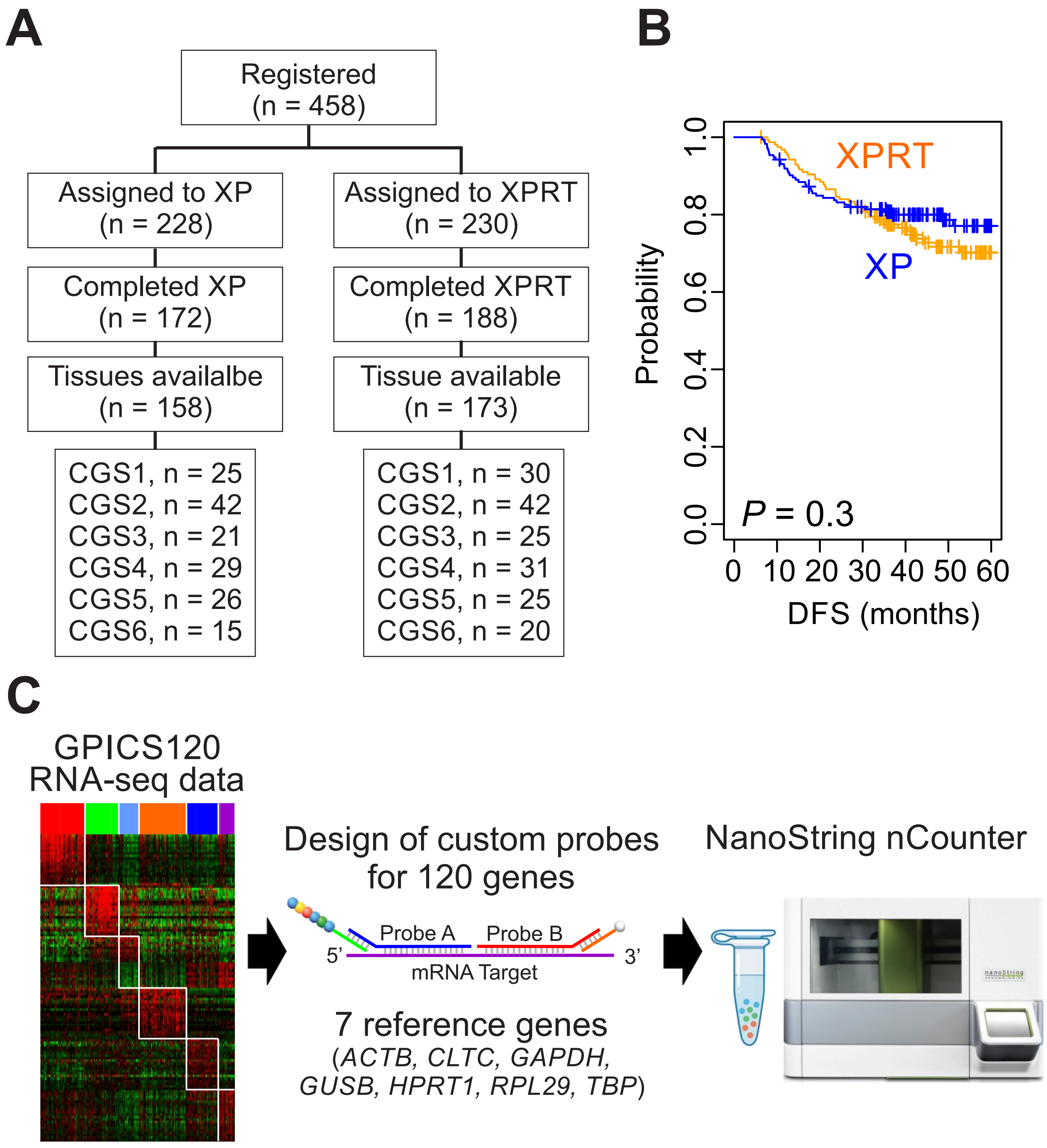

Supplementary Figure S16. Selection and prognosis of patients in ARTIST cohort.

A, Flow chart depicting selection of patients in ARTIST cohort for applying GPICS120 and number of patients with each consensus subtype of gastric cancer.
B, Disease-free survival (DFS) of patients (n = 331) included in GPICS120 analysis by type of adjuvant therapy received. XP, capecitabine and cisplatin; XPRT, XP + concurrent radiotherapy.
C, Schematic diagram for design of custom probes and generation of gene expression data from ARTIST tumor tissues with the NanoString nCounter platform.

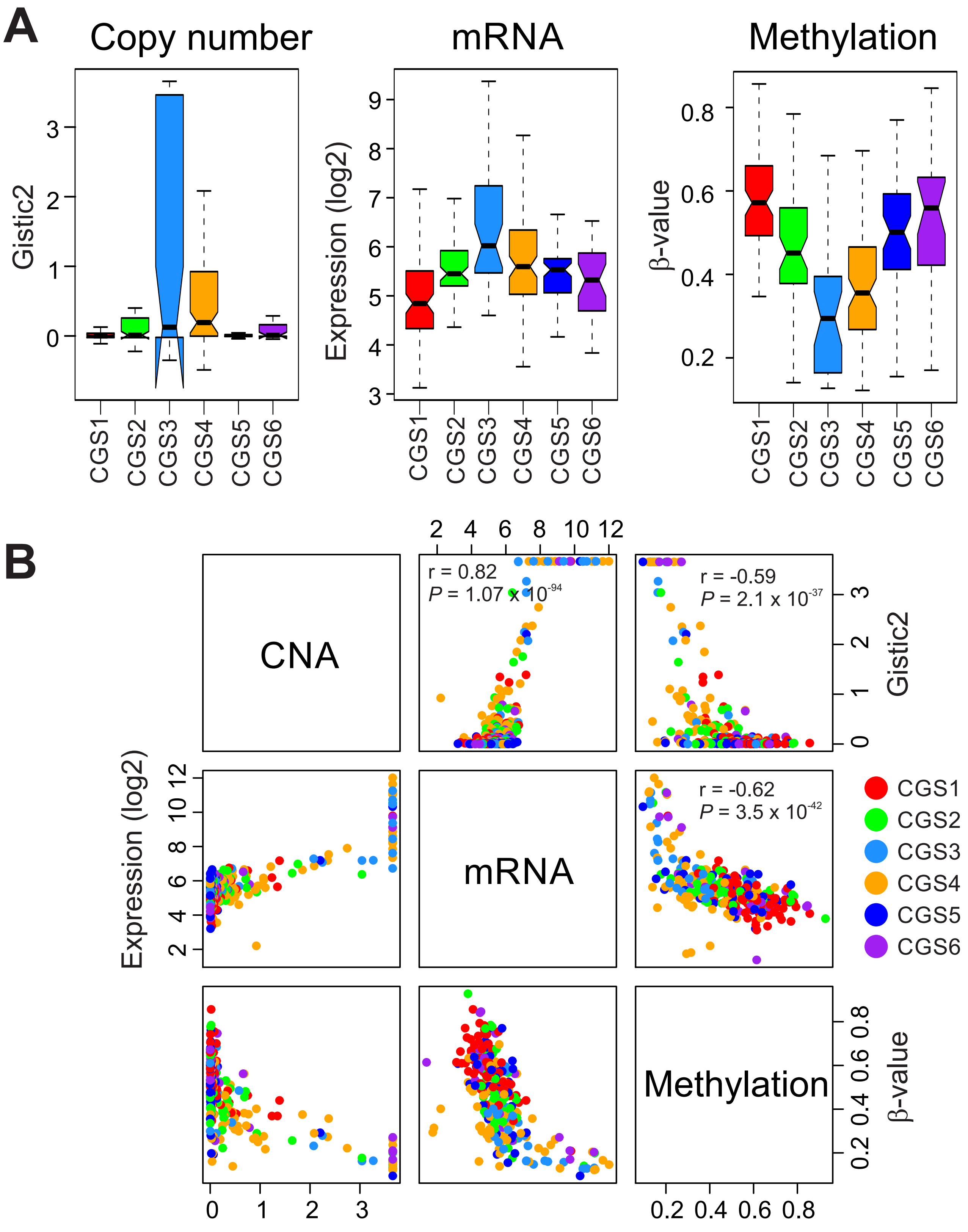

Supplementary Figure S17. HER2 expression is associated with CGS3 subtype of GAC.
A, Copy number alteration, mRNA expression, and promoter methylation of HER2 in consensus subtypes of GAC in TCGA cohort.
B, Scatter plots of HER2 copy number alteration (CNA), mRNA expression, and promoter methylation in TCGA cohort. The significance is estimated by Pearson correlation coefficient.

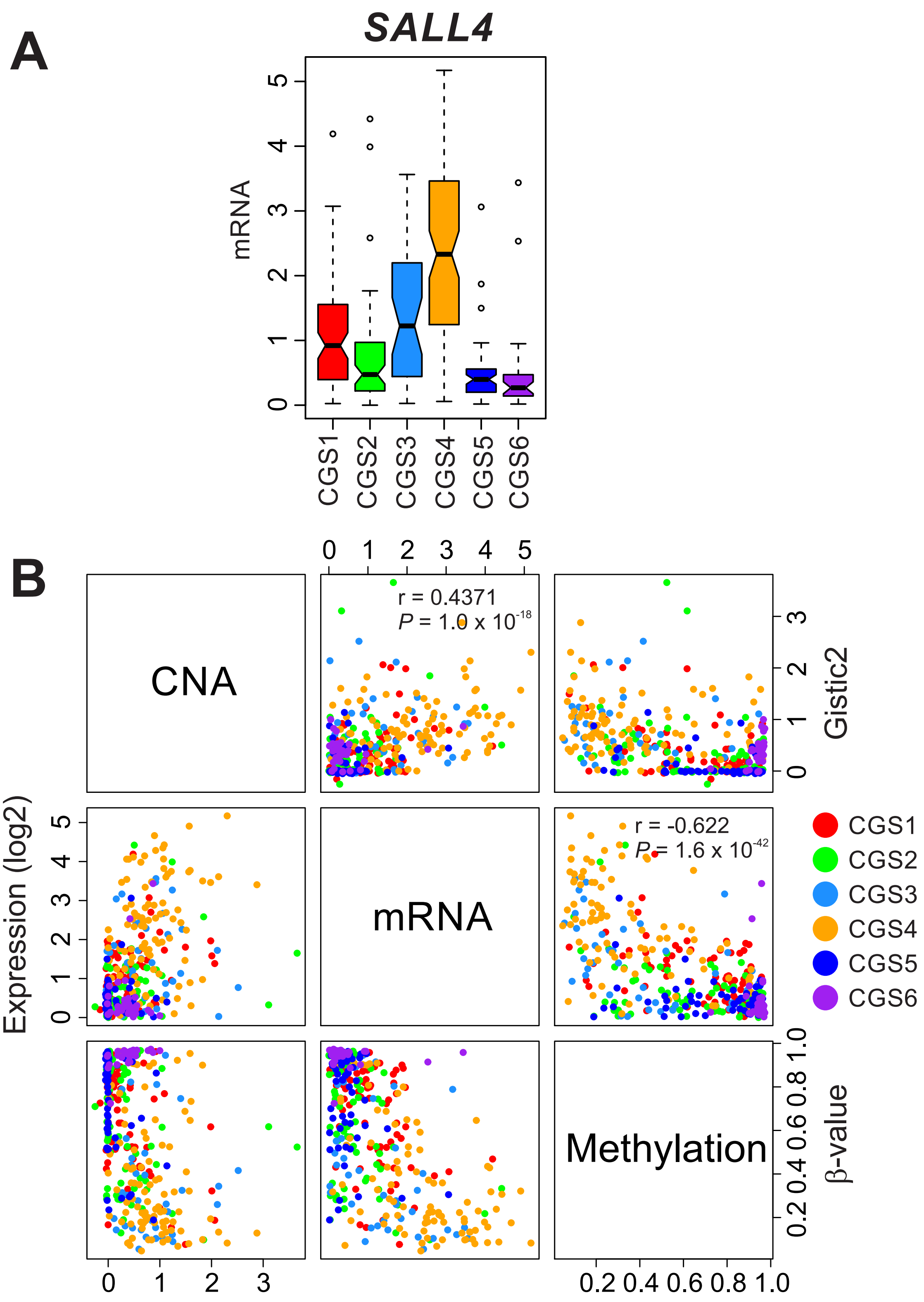

Supplementary Figure S18. SALL4 amplification is associated with CGS4 subtype of GAC.
A, SALL4 mRNA expression in consensus subtypes of GAC in TCGA cohort.
B, Scatter plots of SALL4 copy number alteration (CNA), mRNA expression, and promoter methylation in TCGA cohort. The significance is estimated by Pearson correlation coefficient.

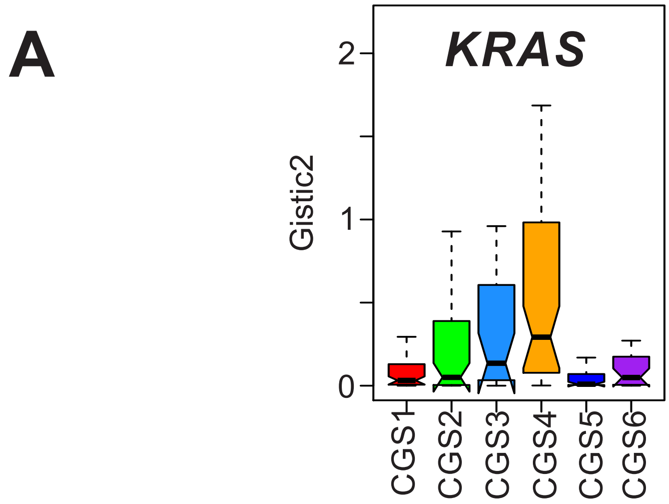

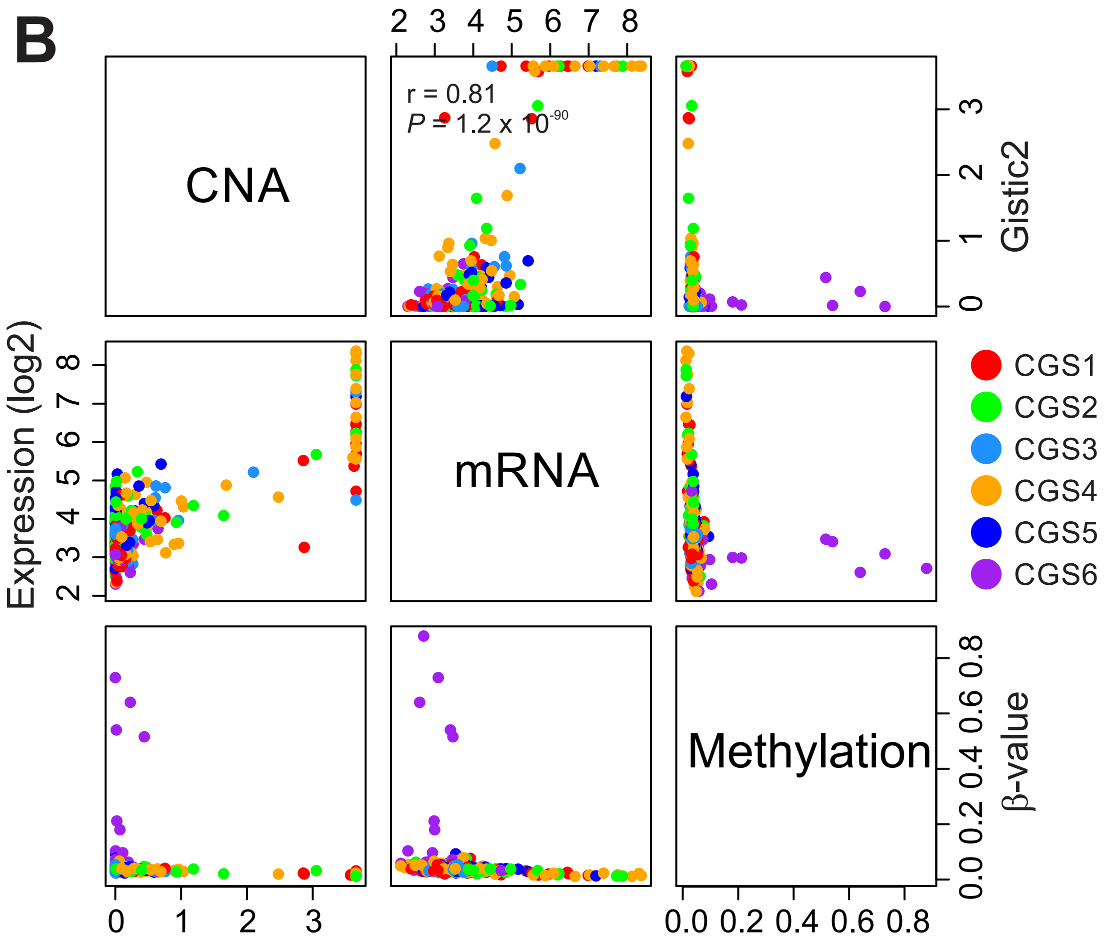

Supplementary Figure S19. KRAS amplification is associated with CGS4 subtype of GAC.
A, KRAS amplification in consensus subtypes of GAC in TCGA cohort.
B, Scatter plots of KRAS copy number alteration (CNA), mRNA expression, and promoter methylation in TCGA cohort. The significance is estimated by Pearson correlation coefficient.

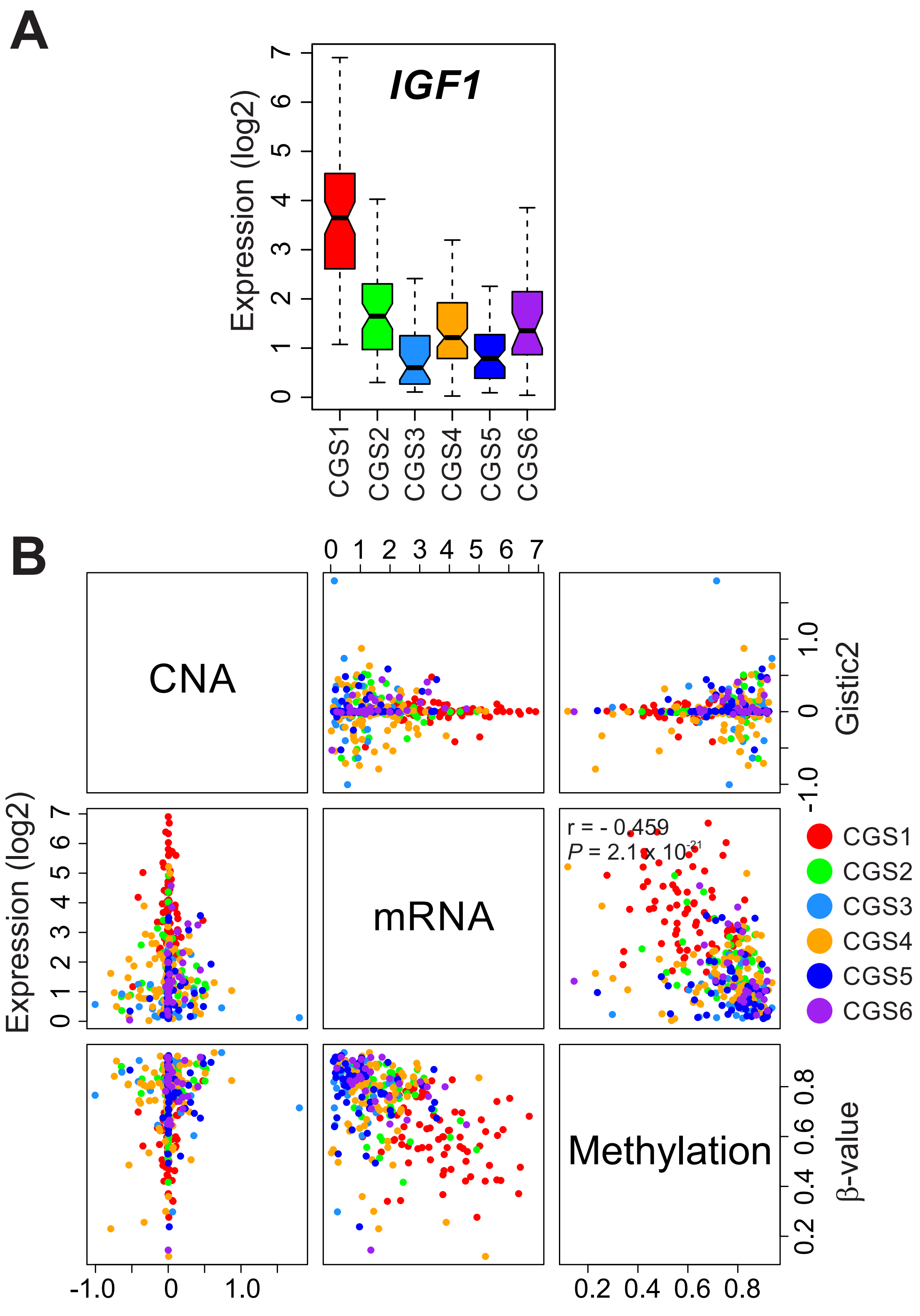

Supplementary Figure S20. IGF1 expression is associated with CGS1 subtype of GAC.
A, IGF1 expression in consensus subtypes of GAC in TCGA cohort.
B, Scatter plots of IGF1 copy number alteration (CNA), mRNA expression, and promoter methylation in TCGA cohort. The significance is estimated by Pearson correlation coefficient.

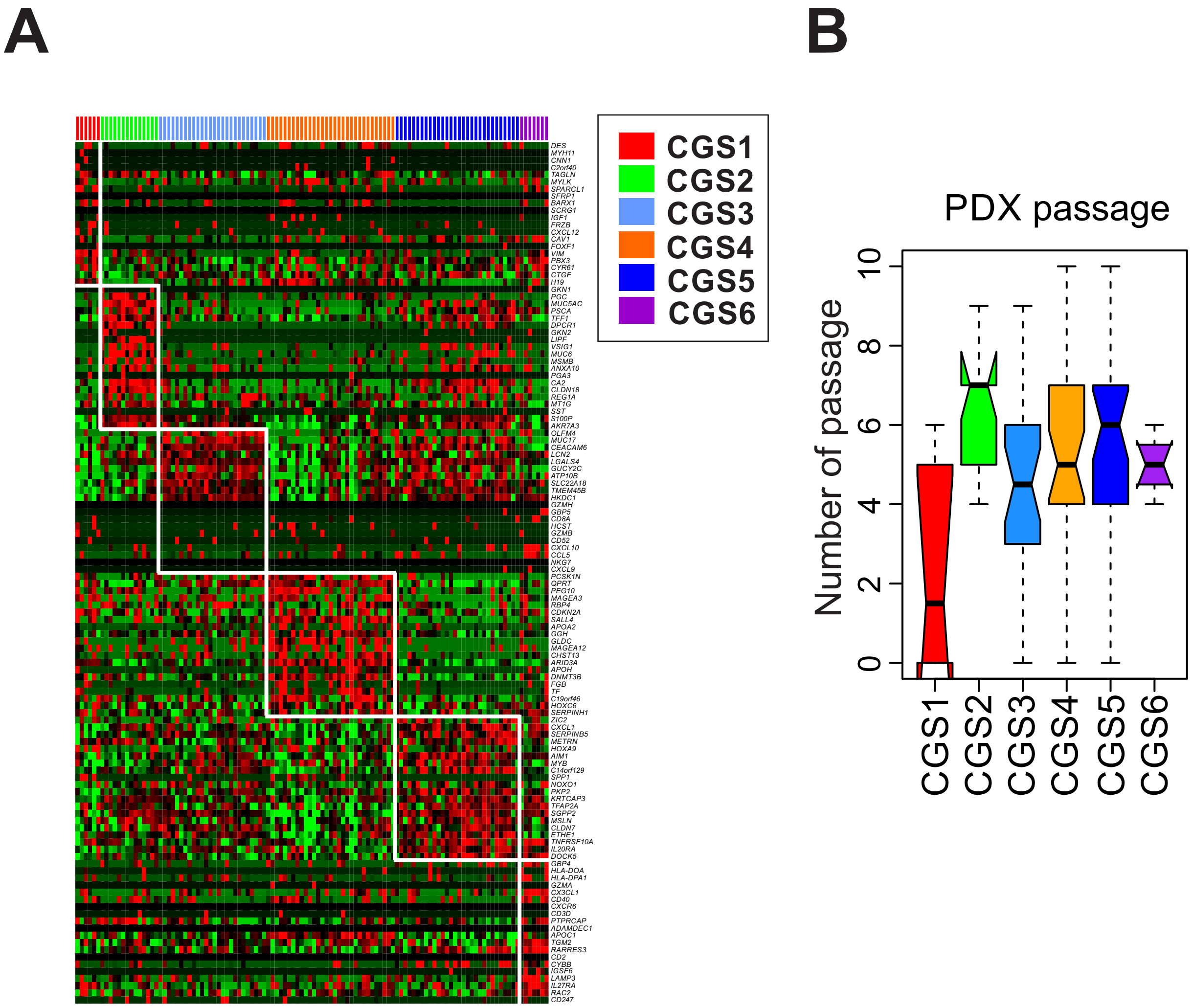

Supplementary Figure S21. Consensus subtypes of GAC in PDX models

A, Expression patterns of GPICS120 genes in gastric cancer PDX tumors. PDX tumors (n = 114) were stratified according to GPICS120. Subtype-specific gene expression patterns were well conserved in the PDX models.
B, Passage number of PDX tumors for each consensus subtype. The subtypes' PDX passage numbers did not differ significantly. The boundaries of each box indicate the 25th to 75th percentile, and the black line within the box marks the mean. Whiskers above and below the box indicate the 10th and 90th percentiles.

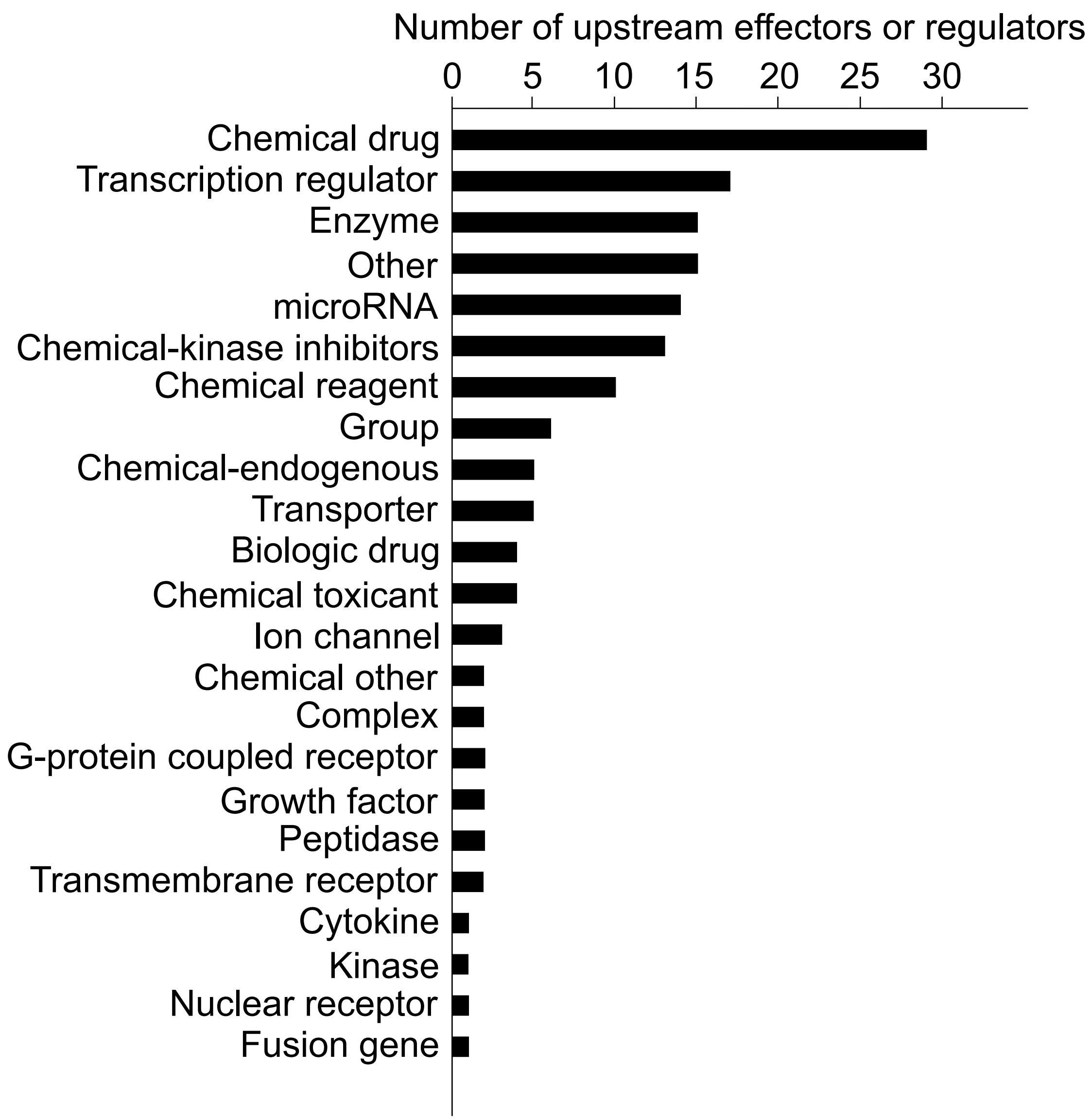

Supplementary Figure. S22. Categories of upstream effectors or regulators sharing gene networks with CGS3 subtype.

Ingenuity Pathway Analysis was performed for CGS3-specific genes (n = 1188) to find potential upstream regulators or effectors sharing networks of genes with the CGS3 subtype. Predicted upstream effectors with significant activation (z-score > 2) were selected for analysis.

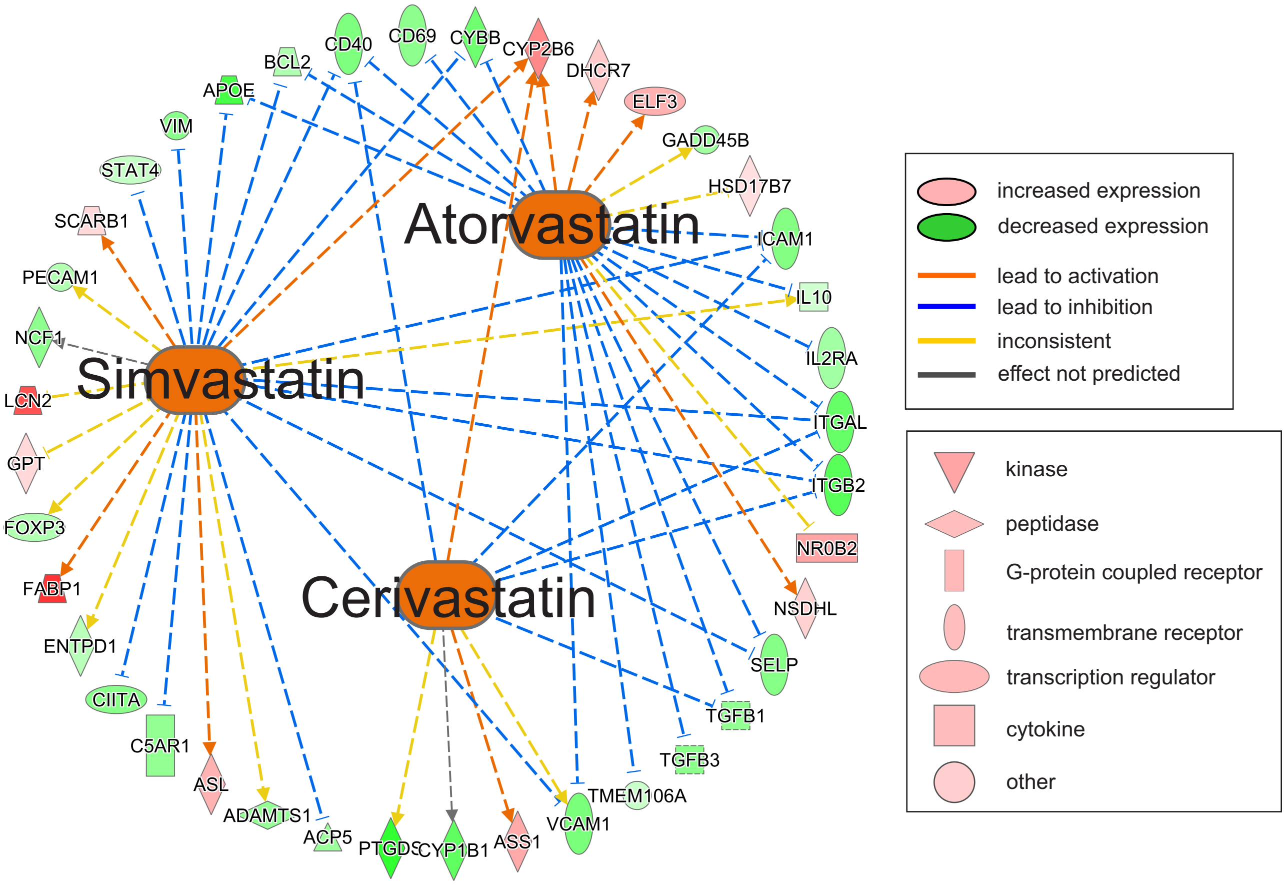

Supplementary Figure S23. Upstream effectors of CGS3-specific genes

Upregulated and downregulated genes in the CGS3 subtype are indicated by red and green, respectively. The lines and arrows represent functional and physical interactions with statin drugs and directions of regulation.

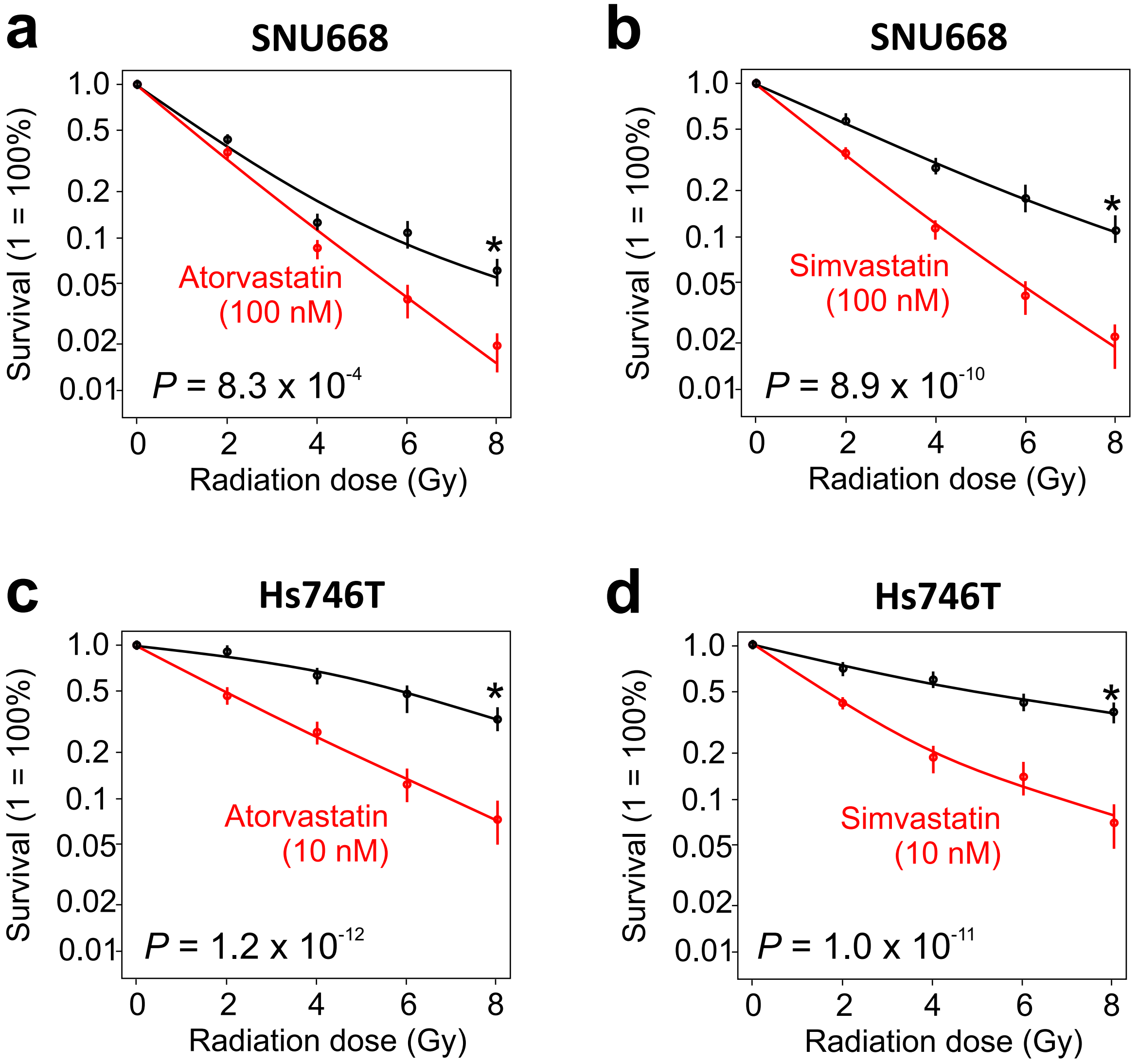

Supplementary Figure S24. Increased sensitivity to ionizing radiation with statins.

Colony-counting assays showed that atorvastatin and simvastatin treatment both sensitized SNU668 and Hs746T non-CGS3 gastric cancer cells to radiation. Seeded cells were exposed to ionizing radiation over the range of 0 to 8 Gy as indicated. P-values indicate the significance of the differences at the 8-Gy dose in each experiment. Means (± SEM) of at least 3 experiments are shown.

Supplementary Table S1. Genomic data used in study

Training set	Platform	Number of patients	Clinical data
Korea	Illumina V3	267	Yes
Samsung	Illumina V3	432	Yes
TCGA	RNA-seq	384	Yes
Shanxi	Affymetrix U133A	134	Yes
Ruijin	Affymetrix U133plus 2	111	No
Singapore1	Illumina V3	99	No
Total		1427	

Validation set			
ACRG	Affymetrix U133plus 2	300	Yes
KNCC	Affymetrix U133A2	167	Yes
Yonsei	Illumina V3	433	Yes
Singapore2	Affymetrix U133plus 2	200	Yes
Total		1100	

TCGA, The Cancer Genome Atlas

ACRG, Asian Cancer Research Group

KNCC, Korea National Cancer Center

Supplementary Table S2. Genomic subtypes of gastric cancer used for COCA analysis.

Name	Characteristics	References
Intrinsic Subtype	Histology	10
Hippo	YAP1 activity	11
TCGA4	Genomic features	6,12
TRS	Recurrence	12
ACRG	Genomic features	9
Yonsei	mRNA expression	8
LNC6	LncRNA expression	13
MP	Mesenchymal-like	7

YAP1, Yes associated protein 1

TCGA, The Cancer Genome Atlas

TRS, TCGA risk score

ACRG, Asian Cancer Research Group

LNC, LncRNA

MP, Mesenchymal phenotype

Supplementary Table S3. Chemical drugs sharing gene networks with CGS3 subtypes

Compounds sharing gene networks	Predicted State	z-score	p-value
**N-acetyl-L-cysteine**	Activated	4.042	1.18E-06
**aspirin**	Activated	3.85	4.21E-07
**fingolimod**	Activated	3.756	2.91E-11
**mifepristone**	Activated	3.59	6.46E-07
**Atorvastatin***	Activated	3.572	0.0011
**geldanamycin**	Activated	3.551	0.0393
**resveratrol**	Activated	3.117	0.000132
**halofuginone**	Activated	2.95	1.27E-05
**propylthiouracil**	Activated	2.891	0.0417
**pentoxifylline**	Activated	2.621	0.0037
**epigallocatechin-gallate**	Activated	2.469	0.00241
**tempol**	Activated	2.425	0.0118
**Y 27632**	Activated	2.425	0.175
**sunitinib**	Activated	2.414	0.0039
**ibrutinib**	Activated	2.412	0.000135
**asoprisnil**	Activated	2.36	4.02E-05
**sulforafan**	Activated	2.345	0.0932
**fenofibrate**	Activated	2.322	0.0107
**eplerenone**	Activated	2.213	0.0254
**thalidomide**	Activated	2.2	0.0105
**Simvastatin***	Activated	2.174	1.93E-05
**captopril**	Activated	2.138	0.0368
**dexamethasone**	Activated	2.135	8.09E-35
**Cerivastatin***	Activated	2.107	0.00387
**fluticasone propionate**	Activated	2.059	9.48E-23
**caffeic acid phenethyl ester**	Activated	2.049	0.00232
**quercetin**	Activated	2.01	0.316
**fenebrutinib**	Activated	2	7.53E-05
**bempedoic acid**	Activated	2	0.00273

P-value indicates significance of overlap between dataset genes and known targets regulated by a compounds. The activation z-score indicates likely activation states of regulators (compounds) based on comparison with a model that assigns random regulation directions.
